# Mechanisms of tethering and cargo transfer during epididymosome-sperm interactions

**DOI:** 10.1186/s12915-019-0653-5

**Published:** 2019-04-18

**Authors:** Wei Zhou, Simone J. Stanger, Amanda L. Anderson, Ilana R. Bernstein, Geoffry N. De Iuliis, Adam McCluskey, Eileen A. McLaughlin, Matthew D. Dun, Brett Nixon

**Affiliations:** 1 Priority Research Centre for Reproductive Science, School of Environmental and Life Sciences, Discipline of Biological Sciences, The University of Newcastle, University Drive, Callaghan, NSW 2308 Australia; 2 Priority Research Centre for Chemical Biology, School of Environmental and Life Sciences, The University of Newcastle, Callaghan, NSW 2308 Australia; 3 School of Biological Sciences, University of Auckland, Auckland, 1142 New Zealand; 4 Faculty of Science and Technology, University of Canberra, Bruce, ACT 2617 Australia; 5 School of Biomedical Sciences and Pharmacy, Faculty of Health and Medicine, The University of Newcastle, Callaghan, NSW 2308 Australia; 6 Hunter Medical Research Institute, Cancer Research Program, New Lambton Heights, NSW 2305 Australia

**Keywords:** Dynamin, Epididymis, Epididymosome, Extracellular vesicle, Exosome, Intercellular trafficking, Membrane raft, Spermatozoa, Sperm maturation

## Abstract

**Background:**

The mammalian epididymis is responsible for the provision of a highly specialized environment in which spermatozoa acquire functional maturity and are subsequently stored in preparation for ejaculation. Making important contributions to both processes are epididymosomes, small extracellular vesicles released from the epididymal soma via an apocrine secretory pathway. While considerable effort has been focused on defining the cargo transferred between epididymosomes and spermatozoa, comparatively less is known about the mechanistic basis of these interactions. To investigate this phenomenon, we have utilized an in vitro co-culture system to track the transfer of biotinylated protein cargo between mouse epididymosomes and recipient spermatozoa isolated from the caput epididymis; an epididymal segment that is of critical importance for promoting sperm maturation.

**Results:**

Our data indicate that epididymosome-sperm interactions are initiated via tethering of the epididymosome to receptors restricted to the post-acrosomal domain of the sperm head. Thereafter, epididymosomes mediate the transfer of protein cargo to spermatozoa via a process that is dependent on dynamin, a family of mechanoenzymes that direct intercellular vesicle trafficking. Notably, upon co-culture of sperm with epididymosomes, dynamin 1 undergoes a pronounced relocation between the peri- and post-acrosomal domains of the sperm head. This repositioning of dynamin 1 is potentially mediated via its association with membrane rafts and ideally locates the enzyme to facilitate the uptake of epididymosome-borne proteins. Accordingly, disruption of membrane raft integrity or pharmacological inhibition of dynamin both potently suppress the transfer of biotinylated epididymosome proteins to spermatozoa.

**Conclusion:**

Together, these data provide new mechanistic insight into epididymosome-sperm interactions with potential implications extending to the manipulation of sperm maturation for the purpose of fertility regulation.

**Electronic supplementary material:**

The online version of this article (10.1186/s12915-019-0653-5) contains supplementary material, which is available to authorized users.

## Background

Mammalian spermatozoa acquire motility and the potential to fertilize an ovum during their descent through the epididymis, an exceptionally long and highly regionalized tubule that connects the testis to the vas deferens. A distinctive hallmark of this process of functional maturation is that it proceeds in the complete absence of de novo gene transcription or protein translation. Rather, it is driven exclusively via extrinsic factors that sperm encounter within the luminal microenvironment of the epididymal tubule. Key elements of this environment are epididymosomes, small membrane bound vesicles that are released from the surrounding epididymal soma via an apocrine secretory pathway [1, 2]. These entities not only protect their encapsulated cargo against the potentially deleterious luminal microenvironment, but also provide a mechanism to affect the bulk delivery of this cargo to maturing spermatozoa. It is therefore not surprising that epididymosomes have been implicated in the trafficking of a broad range of enzymes, structural proteins, chaperones, cytokines, and immunological proteins [3–6], which collectively contribute to sperm function, protection, and subsequent storage prior to ejaculation. In a similar context, epididymosomes have recently begun to attract considerable attention as vehicles for the delivery of alternative cargo, including small non-coding RNA (sncRNA), to spermatozoa and to epididymal epithelial cells situated downstream of their site of secretion [7–10].

It follows that an understanding of the mechanisms by which epididymosomes are targeted to, and interact with, their recipient cells is of fundamental importance to the field of reproductive biology as well as those seeking to resolve the pathway(s) by which paternal exposures alter the sperm epigenome [11]. A defining feature of epididymosome-sperm interactions is their apparent specificity. Thus, in model species such as the bovine, at least two heterogeneous populations of epididymosomes have been characterized, with each possessing the ability to differentiate their investment between live and dead spermatozoa [12]. One such population are defined by their smaller diameter (~ 10–100 nm) and an abundance of CD9, a tetraspanin that decorates the surface of not only epididymosomes, but also the exosomes released from a variety of non-reproductive tissues [13]. This sub-class of epididymosomes displays preferential interaction with live spermatozoa, thus implicating them in sperm maturation/storage [14]. The alternative epididymosome population lack CD9, but feature an abundance of epididymal sperm binding protein 1 and a propensity to interact with dead spermatozoa. This latter population may therefore be involved in protecting viable spermatozoa from degradation products released from dead cells [15]. The selective nature of these interactions suggests that the adherence of epididymosomes to spermatozoa and the subsequent delivery of their encapsulated cargo are tightly regulated events. This model agrees with evidence that epididymosomes isolated from different epididymal segments possess discrete proteomic [16], lipid [16, 17], and sncRNA profiles [7, 9, 10] and may thus be responsible for sequential modification of the macromolecular composition of the sperm cells they encounter. It also agrees with evidence that, as recipient cells for epididymosome cargo, spermatozoa present a number of unique characteristics not typically found in somatic cell populations. Not the least of these are a highly polarized morphology and specialized membrane architecture. Thus, mature sperm cells possess three distinct domains, the (i) head, involved in sperm-oocyte interaction; (ii) mid-piece, which houses the mitochondria and therefore contributes to the cell’s metabolic demands; and (iii) flagellum, which facilitates sperm movement. Differences in membrane protein and lipid composition provide the basis for further subdivision of the sperm head surface topology into the apical and the post-acrosomal (overlying the post-acrosomal sheath) plasma membrane domains.

Previous work has revealed that epididymosomes appear to preferentially interact with the post-acrosomal domain of the sperm head [18, 19]. However, the mechanism(s) by which such selectivity is mediated remain to be fully resolved. Current evidence implicates the involvement of a variety of proteinaceous receptors and their complementary ligands [18]. In this context, our recent studies have identified milk fat globule-EGF factor 8 protein (MFGE8) as a potential ligand for epididymosome-sperm interaction. Accordingly, ultrastructural analyses confirmed the localization of MFGE8 on the epididymosome surface extending into stalk-like projections associated with sites of epididymosome-sperm interaction. Furthermore, antibody masking of MFGE8 ligands compromised the efficiency of epididymosome-mediated protein transfer to recipient spermatozoa [19]. Downstream of this initial adhesion event, it has been postulated that the epididymosome and sperm membranes undergo a transient fusion leading to delivery of the epididymosome cargo and a subsequent detachment of the epididymosome [20, 21]. Although several membrane-trafficking protein families have been identified within the epididymosome proteome [3, 16, 22], there remains a dearth of evidence concerning their precise functional roles. Similarly, specialized microdomains known as membrane or lipid rafts [23] have also been shown to play a role in coordinating the initial docking of sperm and epididymosome membranes [24], an interaction that results in the direct transfer of a subset of epididymosome raft-associated (i.e., glycosylphosphatidylinositol-linked) proteins into the cognate raft domains of the maturing sperm cell [2]. It is also conceivable that lipid raft microdomains could facilitate the sequestration of complementary receptors/ligands and downstream fusion machinery within the respective epididymosome and sperm membranes to enable transfer of non-raft proteins. While this latter model of epididymosome-sperm interaction draws analogy with similar interactions recorded between exosomes and recipient somatic cells, much of the mechanistic detail remains elusive. This is particularly the case in species such as the mouse in which there are currently only limited reports of epididymosome characterization. In seeking to address this paucity of knowledge, here we report the use of an in vitro co-culture system to track the transfer of biotinylated protein cargo between mouse epididymosomes and recipient spermatozoa from the caput epididymis; the most active epididymal segment in terms of protein secretion and one that is of critical importance for promoting sperm maturation [21]. Moreover, we have utilized pharmacological inhibition strategies in an effort to characterize key elements of the sperm proteome responsible for the selective uptake of epididymosome cargo.

## Results

### Mouse caput epididymosomes selectively transfer biotinylated proteins to the head and mid-piece of homologous spermatozoa

**Fig. 1:**
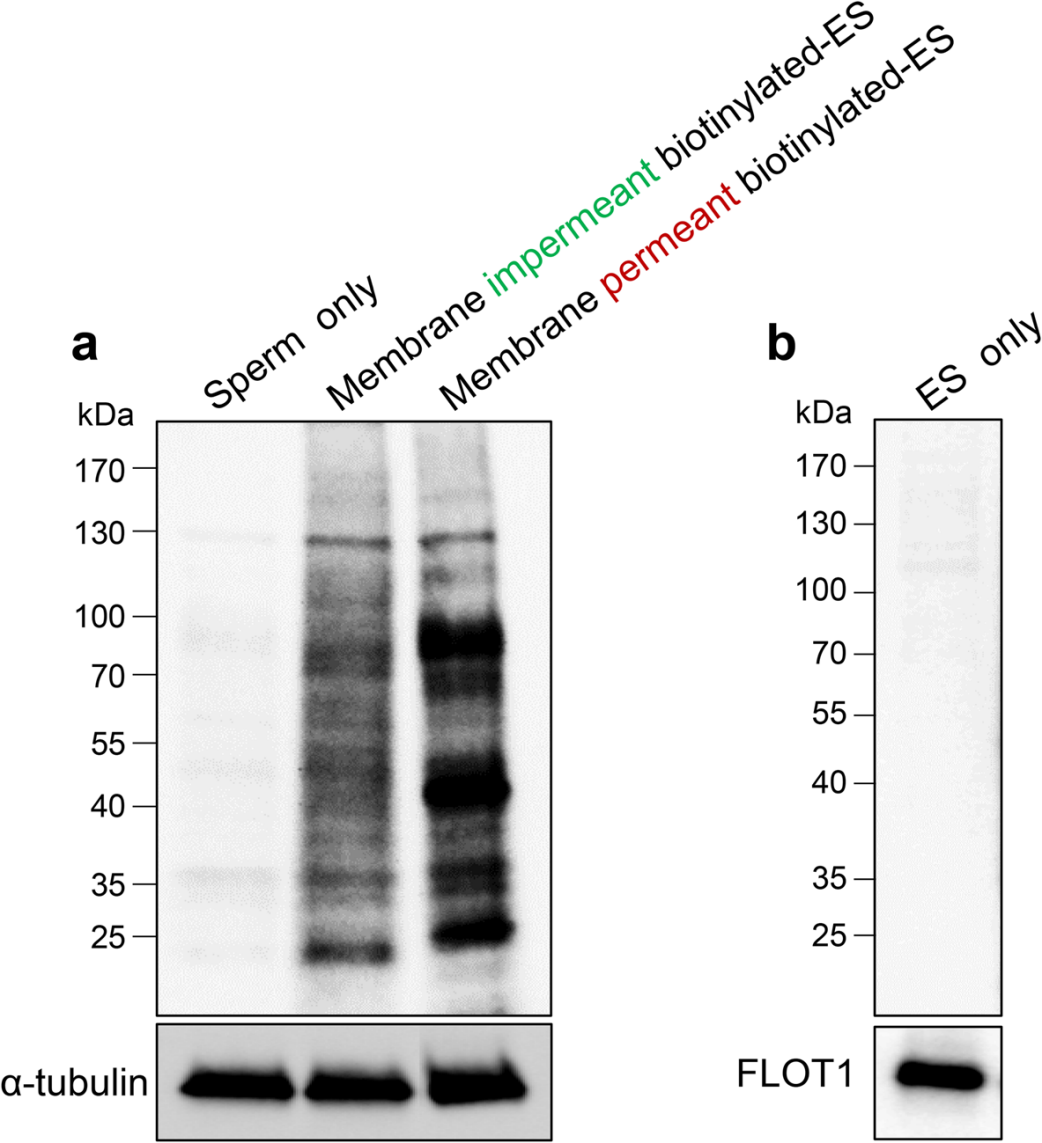
Mouse epididymosomes mediate the transfer of biotinylated proteins to homologous spermatozoa. **a** Epididymosomes were labeled with membrane impermeant or membrane permeant biotin reagents (i.e., sulfo-NHS-LC-biotin and BMCC-biotin, respectively) before being co-cultured with spermatozoa in vitro for 1 h. After incubation, spermatozoa were solubilized and lysates prepared for Western blotting and affinity labeling with HRP-conjugated streptavidin to assess the incorporation of biotinylated epididymosome cargo into their proteome. To demonstrate the selectivity of the transfer process, equivalent lysates from naïve populations of sperm that remained unexposed to epididymosomes were resolved alongside the treatment groups (Sperm only), revealing minimal endogenously biotinylated proteins. Blots were stripped and re-probed with α-tubulin to confirm an equal quantity of protein was loaded in each lane. **b** Unlabeled populations of epididymosomes (ES only) were also subjected to Western blotting, revealing no endogenously biotinylated proteins. The presence of proteins in this blot was affirmed by re-probing with the epididymosome marker FLOT1. These experiments were repeated three times and representative blots are shown

To begin to characterize the molecular mechanisms underpinning mouse epididymosome-sperm interactions, we employed a previously optimized co-culture system [7] to track the transfer of biotinylated protein cargo to spermatozoa. For this purpose, both membrane impermeant (sulfo-NHS-LC-biotin) and membrane permeant (BMCC-biotin) reagents were applied to label the caput epididymosome proteome prior to their co-culture with isolated populations of caput spermatozoa. Thereafter, the efficacy of biotinylated protein transfer was assessed via affinity labeling of sperm lysates with streptavidin-HRP, revealing a substantive delivery of proteins ranging in size from ~ 15–150 kDa. As anticipated on the basis of their differential targeting of membrane vs whole epididymosome proteins, and reactivity toward primary amine (sulfo-NHS-LC-biotin) vs sulfhydryl groups (BMCC-biotin), the profile of biotinylated epididymosome proteins transferred to the spermatozoa displayed marked differences (Fig. 1a). Indeed, as anticipated based on its propensity to label both membrane and encapsulated protein cargo, substantially more of the membrane permeant biotin appeared to be transferred to spermatozoa (Fig. 1a). These data confirm that caput epididymal spermatozoa are readily able to incorporate epididymosome proteins into their proteome after even a relatively brief period (i.e., 1 h) of in vitro co-culture. Attesting to the selectively of this transfer process, we detected minimal endogenously biotinylated proteins within lysates prepared from either naïve populations of spermatozoa that had not encountered epididymosomes or within the epididymosomes themselves (Fig. 1b).

**Fig. 2:**
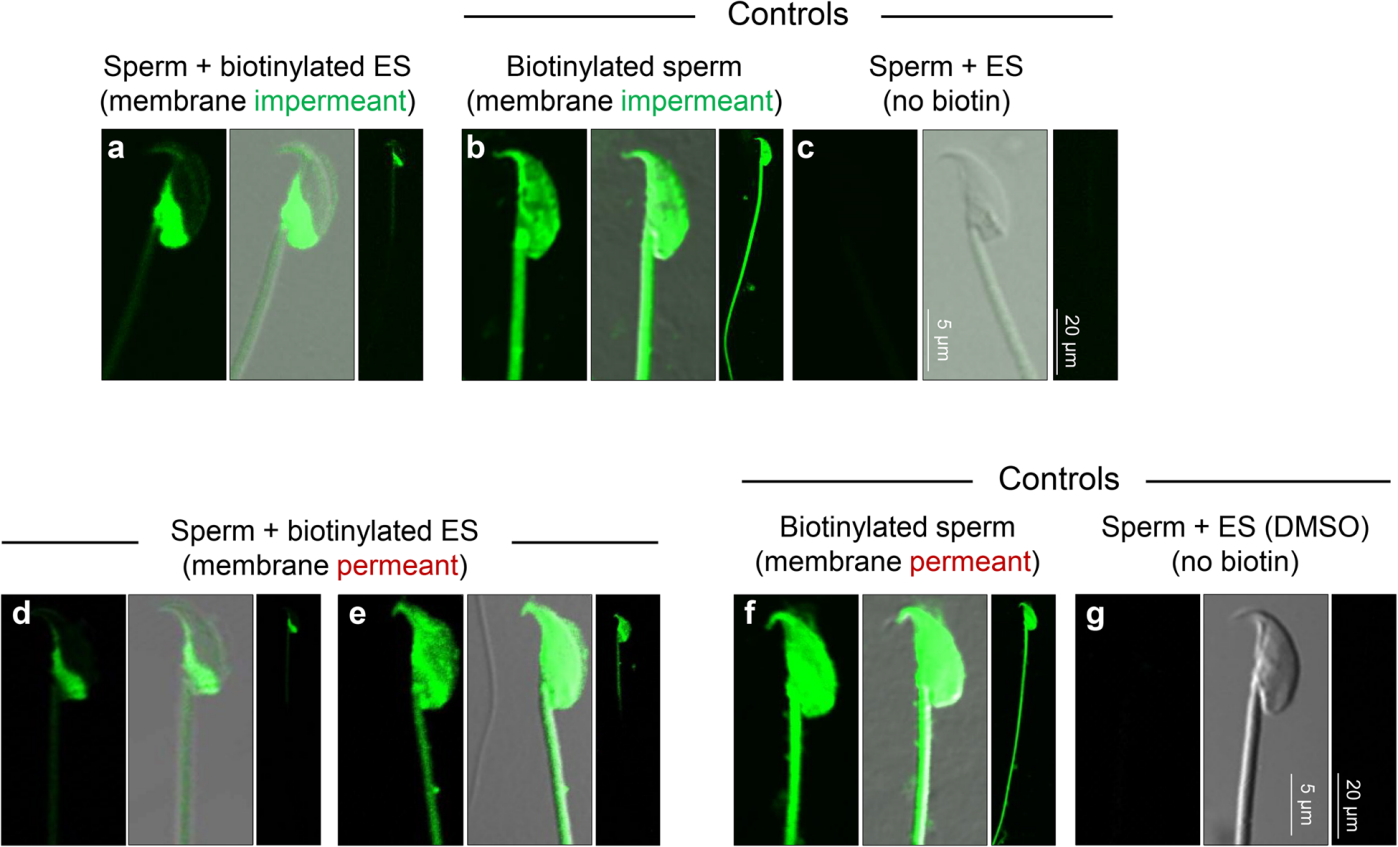
Epididymosomes transfer biotinylated proteins to the head of caput epididymal spermatozoa**.** Mouse caput spermatozoa were co-cultured with biotin-labeled epididymosomes in vitro for 1 h prior to being fixed and labeled with streptavidin conjugated to Alexa Fluor 488 to detect the spatial profile of transferred biotinylated proteins. **a**, **d** Prominent post-acrosomal domain staining was observed for both biotin reagents, while **e** whole head and mid-piece staining was exclusively observed in a small portion of cells incubated with epididymosomes biotinylated with the membrane permeant reagent. Representative controls are included in which spermatozoa were either directly labeled with **b** membrane impermeant biotin or **f** the membrane permeant biotin, both of which yielded a distinct profile of biotinylation across the entire spermatozoon. **c**, **g** Spermatozoa were also incubated with non-biotinylated epididymosomes as a negative control to confirm the absence of endogenous biotin signal under the same imaging conditions. All experiments were repeated at least three times and representative images are shown

Having confirmed that mouse epididymosomes are capable of transferring proteins to caput spermatozoa, we next performed affinity labeling of the cells with streptavidin conjugated to Alexa Fluor 488 to determine the sperm domain(s) to which this cargo was targeted (Fig. 2). This analysis confirmed the selectively of epididymosome-sperm interactions, with the post-acrosomal domain of the sperm head serving as the dominant site for protein uptake after a 1 h co-incubation, irrespective of the biotin-labeling regimen used (Fig. 2a, d). In the case of epididymosomes subjected to membrane impermeant biotinylation (i.e., sulfo-NHS-LC-biotin), additional sperm labeling, albeit far less intense, was detected within the anterior domain of the head and mid-piece of the flagellum (Fig. 2a). Using the alternative pool of epididymosomes labeled with membrane permeant biotin (i.e., BMCC-biotin), ~ 10% of the spermatozoa presented with additional foci of intense labeling throughout the head and extending into the mid-piece of the flagellum (Fig. 2e). To discount the possibility of non-specific labeling, spermatozoa were subjected to direct biotinylation, yielding a distinct pattern of labeling uniformly distributed across all sperm domains (i.e., head and flagellum) (Fig. 2b, f). Additionally, we failed to detect any endogenous biotin signal in sperm incubated with non-biotin-labeled epididymosomes (Fig. 2c, g).

### Caput epididymosome-sperm interactions can be discriminated into sequential adhesion and transient fusion/dispersal of cargo events

**Fig. 3:**
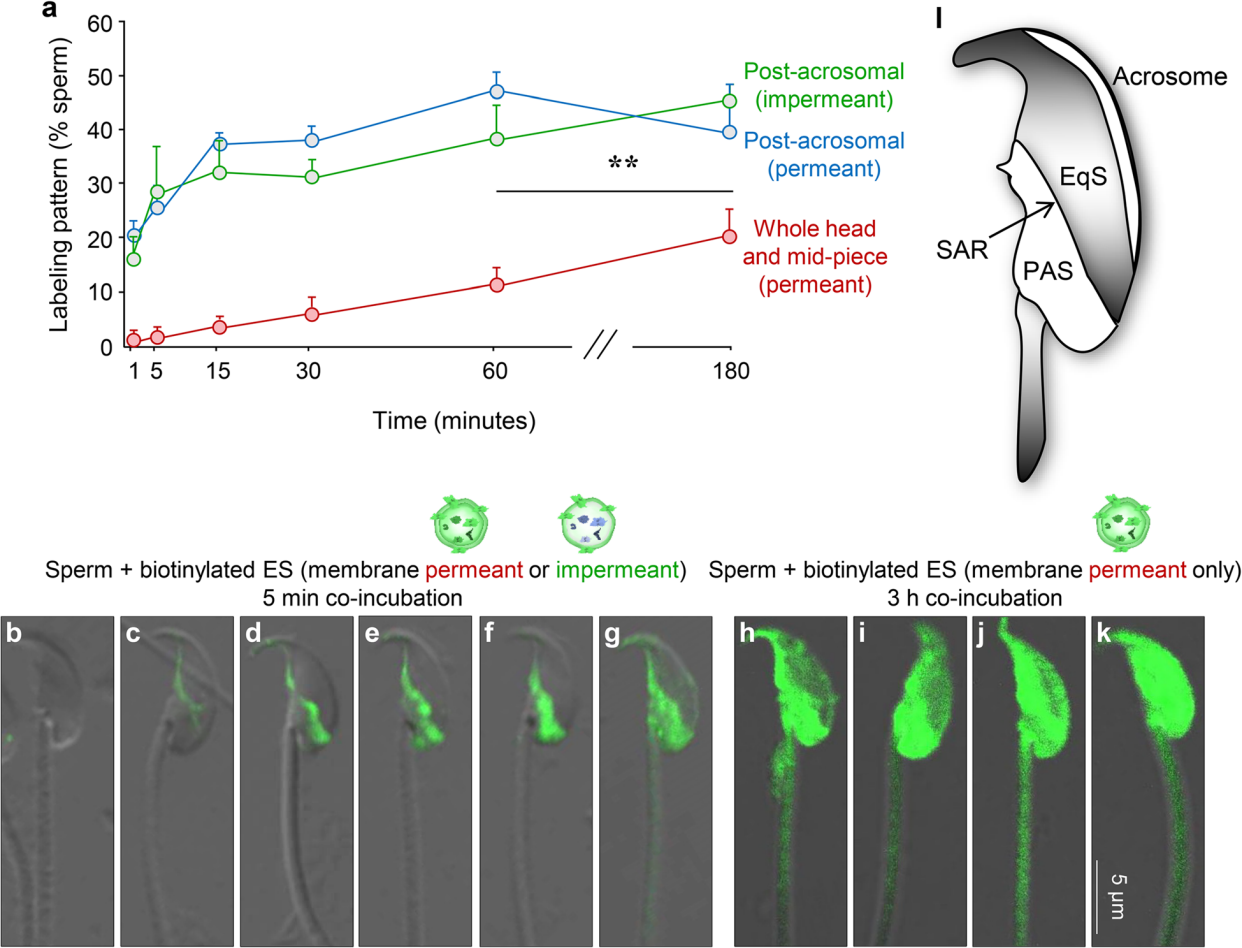
Exploration of the kinetics of epididymosome-sperm interaction. **a** Caput spermatozoa were co-cultured with biotin-labeled epididymosomes and sampled at regular intervals during the course of a 3-h incubation before being subjected to biotin detection. The dominant patterns of biotin localization in the post-acrosomal domain or whole head and mid-piece of the spermatozoa were quantified, with 100 cells being examined per sample (*n* = 3; graphical data are presented as mean ± SEM), **P < 0.01. **b**–**k** Two phases of epididymosome-sperm interaction were distinguished, with an initial rapid uptake of biotin-labeled cargo being detected primarily in the post-acrosomal domain within ≤ 5 min of co-culture. **b**–**g** Fluorescence images representing the different patterns of biotinylated protein transfer detected after a co-incubation period of 5 min are provided. These images depict the gradient of increasing biotin signal, initially being detected in the SAR before extending distally to encompass the entire post-acrosomal domain. **h**–**k** A second phase of interaction was recorded exclusively with the use of membrane permeant biotin and became particularly apparent during extended incubation (i.e., at either 1 h or 3 h of co-culture). Thus, biotin fluorescence in these cells extended over the whole head and mid-piece of the flagellum. Shown are representative images of the patterns of biotinylated protein transfer detected after a co-incubation period of 3 h. **l** Schematic of the structural domains of the mature mouse sperm head; EqS, equatorial segment; SAR, sub-acrosomal ring; PAS, post-acrosomal sheath

In seeking to account for the appearance of additional diffuse localization of membrane permeant (but not impermeant) biotin throughout the sperm head and mid-piece, we elected to study the kinetics of protein transfer between caput epididymosomes and recipient caput spermatozoa. As shown in Fig. 3a, both forms of biotinylated protein were incorporated into the post-acrosomal domain of the sperm head with similar overall kinetics and efficiency. Thus, within as little as 1 min of co-culture, ~ 16–20% of the sperm population displayed positive post-acrosomal domain labeling. Thereafter, the percentage of positively labeled cells continued to gradually increase such that at 1 h of co-culture, ~ 40–50% of the sperm population were characterized by positive post-acrosomal domain labeling (Fig. 3a). In the case of epididymosome proteins labeled with the membrane impermeant (i.e., sulfo-NHS-LC-biotin), the post-acrosomal domain appeared to be their final repository. Indeed, in the majority of cells, membrane impermeant biotin was not detected at appreciable levels in any alternative domains irrespective of extending the duration of co-incubation for up to 3 h (i.e., only 3% of the cells featured whole head and mid-piece labeling after 3 h incubation, data not shown) (Fig. 3a). In marked contrast, spermatozoa incubated with the alternative pool of membrane permeant biotinylated epididymosomes (BMCC-biotin) experienced a reduction in post-acrosomal labeling between 1 and 3 h of co-incubation. This apparent loss of post-acrosomal labeling was accompanied by a reciprocal increase in those cells displaying whole head and mid-piece labeling, effectively doubling to account for ~ 20% of the sperm population at 3 h compared to 10% at 1 h (*P* < 0.01, Fig. 3a).

On the basis of these data, we infer that epididymosome-sperm interactions may encompass an adhesion event (detected by both forms of biotin transfer) followed by transient fusion and dispersal of cargo (detected by the membrane permeant biotin transfer only). To explore the validity of this model, we examined profiles of biotin labeling in spermatozoa isolated during both early (i.e., 5 min; Fig. 3b–g) and late phases (3 h; Fig. 3h–k) of co-incubation with epididymosomes. This analysis identified two putatively discrete stages of sperm-epididymosome interaction. Thus, during the early phases of co-incubation (1 min), biotinylated epididymosome proteins were mainly found to populate the sub-acrosomal ring (SAR; Fig. 3l) of the sperm head (similar to the image presented in Fig. 3c). This labeling pattern was, however, replaced (within 5 min) as the majority of labeled sperm appeared to accumulate biotinylated proteins distally to the point where they eventually occupied the entire post-acrosomal domain (representative images of this gradient of increasing labeling are depicted in Fig. 3d–g). Such staining characteristics were conserved between both forms of biotin utilized in this study. By contrast, the latter phases of co-incubation (3 h) saw a clear differentiation in terms of the fate of the epididymosome proteins. Thus, those proteins labeled with membrane impermeant biotin remained exclusively within the post-acrosomal domain of the sperm head. The alternative sub-population of proteins labeled with membrane permeant biotin proceeded to undergo bidirectional dispersal into both the anterior region of the sperm head (equatorial segment and acrosomal domain) and mid-piece of the flagellum with similar overall kinetics (Fig. 3h–k). These data accord with those documented during our previous application of carboxyfluorescein diacetate succinimidyl ester (CFSE), a dye that we have also tracked from epididymosomes into the sperm head and mid-piece after 3 h co-incubation in vitro [7]. The lack of an equivalent dispersal when using membrane impermeant biotin, suggests this phenomenon may be restricted to proteins encapsulated within, as opposed to on the surface, of epididymosomes.

**Fig. 4:**
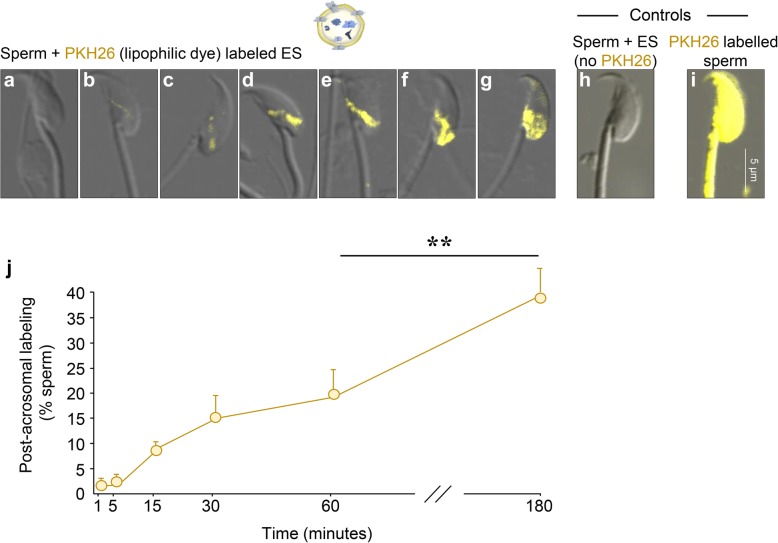
Examination of the transfer of lipophilic dye (PKH26) between epididymosomes and spermatozoa. **a**–**g** Caput spermatozoa were briefly co-cultured (≤ 5 min) with populations of epididymosomes preloaded with the lipophilic dye, PKH26. After incubation, spermatozoa were washed and fixed prior to the analysis of PKH26 labeling profiles via immunofluorescence microscopy. Representative immunofluorescence images of different spermatozoa confirmed the incorporation of PKH26 dye, with staining patterns appearing broadly similar to those documented for proteins labeled with membrane impermeant biotin. That is, PKH26-labeled lipids were predominantly transferred to the SAR and post-acrosomal domain of the sperm head. Representative controls were included in which spermatozoa were either **h** incubated with non-labeled epididymosomes to confirm no auto-fluorescence or alternatively, **i** directly labeled with PKH26 dye (independent of epididymosomes), which resulted in staining of the whole spermatozoon. **j** To examine the kinetics of PKH26 transfer, caput spermatozoa were co-cultured with PKH26-labeled epididymosomes and sampled at regular intervals during the course of a 3-h incubation. The dominant pattern of post-acrosomal labeling of the spermatozoa were quantified, with 100 cells being examined per sample (*n* = 3; graphical data are presented as mean ± SEM). **P < 0.01

We elected to explore this possibility using a lipophilic dye PKH26, which incorporates directly into the membrane bilayer of the epididymosome. Following co-incubation of PKH26-labeled epididymosomes with spermatozoa, we documented a rapid transferal of the dye into the SAR of the sperm head (Fig. 4b–e). Thereafter, intense PKH26 labeling was documented throughout the post-acrosomal domain (Fig. 4f–g). Notably, real-time imaging of live cells confirmed that both the spatial and temporal characteristics of epididymosome-mediated transfer of PKH26 closely approximated those of the biotinylated epididymosome proteins reported above (Additional file 1: Figure S1). An additional foci of PKH26 labeling was also detected within the anterior domain of the head, albeit far less intense than that of the post-acrosomal domain (Fig. 4g and Additional file 1: Figure S1). Moreover, while the initial transfer of PKH26-labeled lipids proceeded slower (Fig. 4j) than that recorded for biotinylated protein transfer (Fig. 3a), the percentage of labeled spermatozoa proved equivalent after 3 h of co-culture. The specificity of epididymosome-mediated transfer of PKH26 labeling was confirmed by the absence of any endogenous fluorescence signals after co-incubation of sperm with non-labeled epididymosomes (Fig. 4h). By contrast, sperm labeled directly with PKH26 (i.e., in the absence of epididymosomes) readily incorporated the dye over their entire surface (Fig. 4i).

### Dynamin mechanoenzymes are implicated in epididymosome-mediated transfer of proteins to caput spermatozoa

**Fig. 5:**
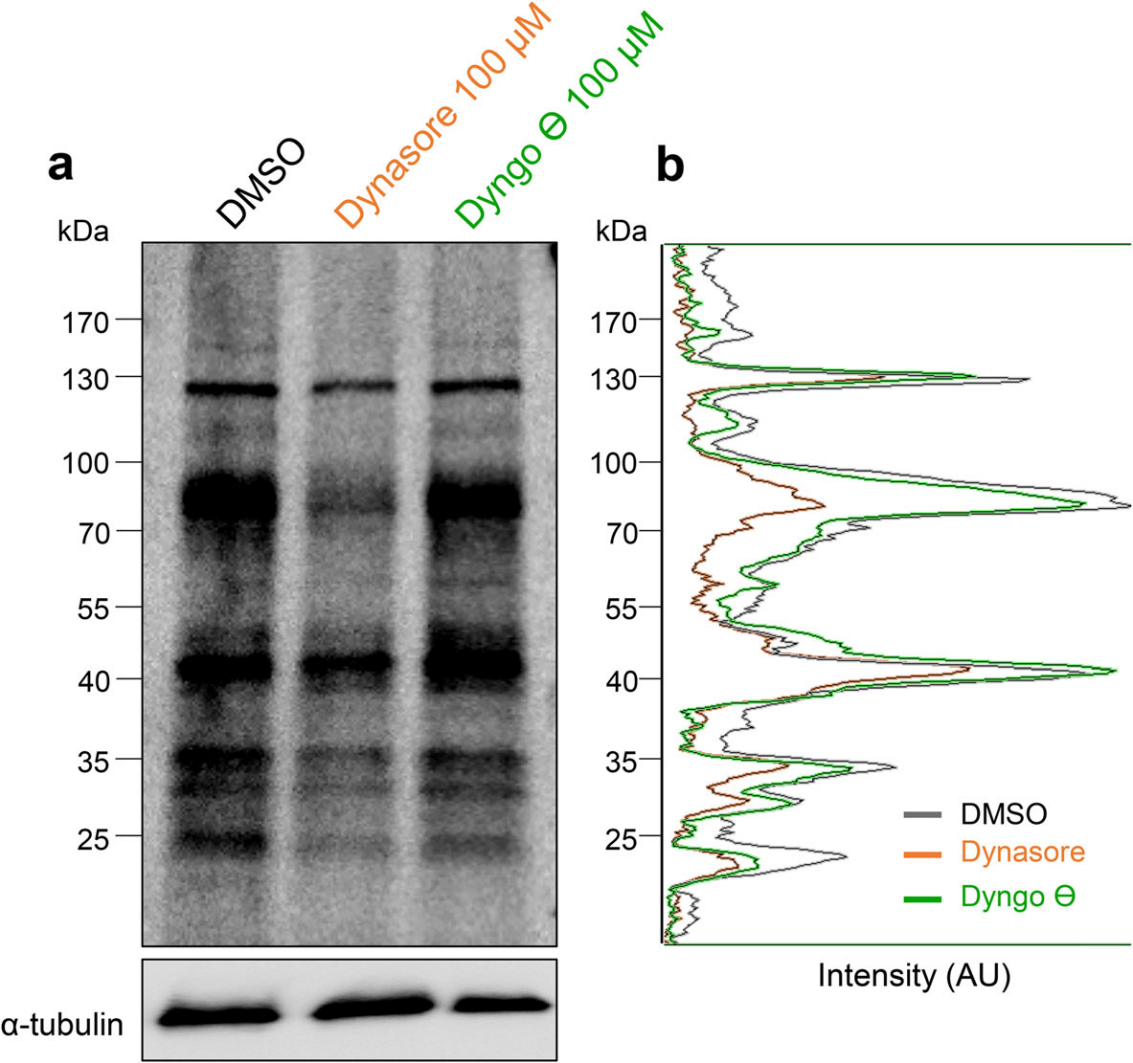
DNM inhibition reduces the transfer of epididymosome protein cargo to spermatozoa in vitro. **a** Spermatozoa were pre-treated with DMSO (vehicle control) or an equivalent concentration (100 μM) of Dynasore or Dyngo-Ɵ (an inactive isoform control) for 30 min, before being subjected to incubation with biotin (membrane permeant)-labeled epididymosomes for 3 h. In this assay, the ratio of epididymosomes to sperm was adjusted such that epididymosomes equating to a single mouse were co-incubated with pooled spermatozoa from four mice. Cell lysates were then subjected to Western blotting to detect the efficacy of biotinylated protein cargo transfer. **b** The pixel intensity of biotinylated protein bands detected within each lane were quantified using ImageJ software, and a representative trace of this analysis is included alongside the blot. **a** After imaging, blots were stripped and re-probed with anti-α-tubulin antibodies to confirm equivalent protein loading. These experiments were replicated three times with each replicate containing pooled sperm lysates from three mice, and a representative blot is presented

In view of the ability to differentiate caput epididymosome-sperm interactions into initial adhesion and downstream transient fusion events, we sought to capitalize on somatic cell literature implicating the dynamin (DNM) family of mechanoenzymes as master regulators of analogous forms of intercellular vesicle trafficking. As a caveat, however, our previous work has established that the dynamin1 (DNM1) and dynamin2 (DNM2) isoforms, which are present in the mouse sperm proteome, appear to concentrate within the anterior acrosomal domain of the mature cell [25], a location seemingly incompatible with the tethering of epididymosomes in the SAR/post-acrosomal domain. We therefore elected to track the efficacy of biotinylated epididymosome protein transfer to populations of spermatozoa that had been pre-incubated with Dynasore, a DNM inhibitor that targets DNM1 and DNM2 with equivalent efficacy, or Dyngo-Ɵ, an inactive isoform control. Since we anticipate that DNM would likely regulate epididymosome fusion, as opposed to adhesion, this study featured the use of membrane permeant biotin reagent for epididymosome labeling and was conducted over an incubation period of 3 h to coincide with protein uptake and dispersal (Fig. 3h–k). This strategy revealed that pharmacological inhibition of DNM1/DNM2 had no discernible impact on the ability of spermatozoa to incorporate biotinylated epididymosome proteins into their post-acrosomal domain, with ~ 40% of the cells displaying this pattern of labeling irrespective of the treatment group from which they originated (i.e., untreated, Dynasore or Dyngo-Ɵ). By contrast, dynamin inhibition effectively halved the number of recipient spermatozoa in which the biotinylated proteins were redistributed throughout the anterior region of the head and into the mid-piece of the tail (down from ~ 20% in the untreated group to ~ 10% in the Dynasore treatment group). Consistent with these data, densitometric quantification confirmed that dynamin inhibition significantly reduced, but did not eliminate, the transfer of biotinylated proteins from epididymosomes to spermatozoa (Fig. 5a, b). Importantly, no such reduction was witnessed in spermatozoa pre-treated with Dyngo-Ɵ, thus precluding the possibility of non-specific pharmacological inhibition. Similarly, neither the DNM inhibitor nor the inactive isoform control had a detrimental impact on spermatozoa viability, which remained above 60% in all treatments.

Having implicated DNM-dependent mechanisms in the transfer of epididymosome proteins to spermatozoa, we next sought a more detailed characterization of DNM localization in the immature population of caput epididymal sperm used throughout this study.

**Fig. 6:**
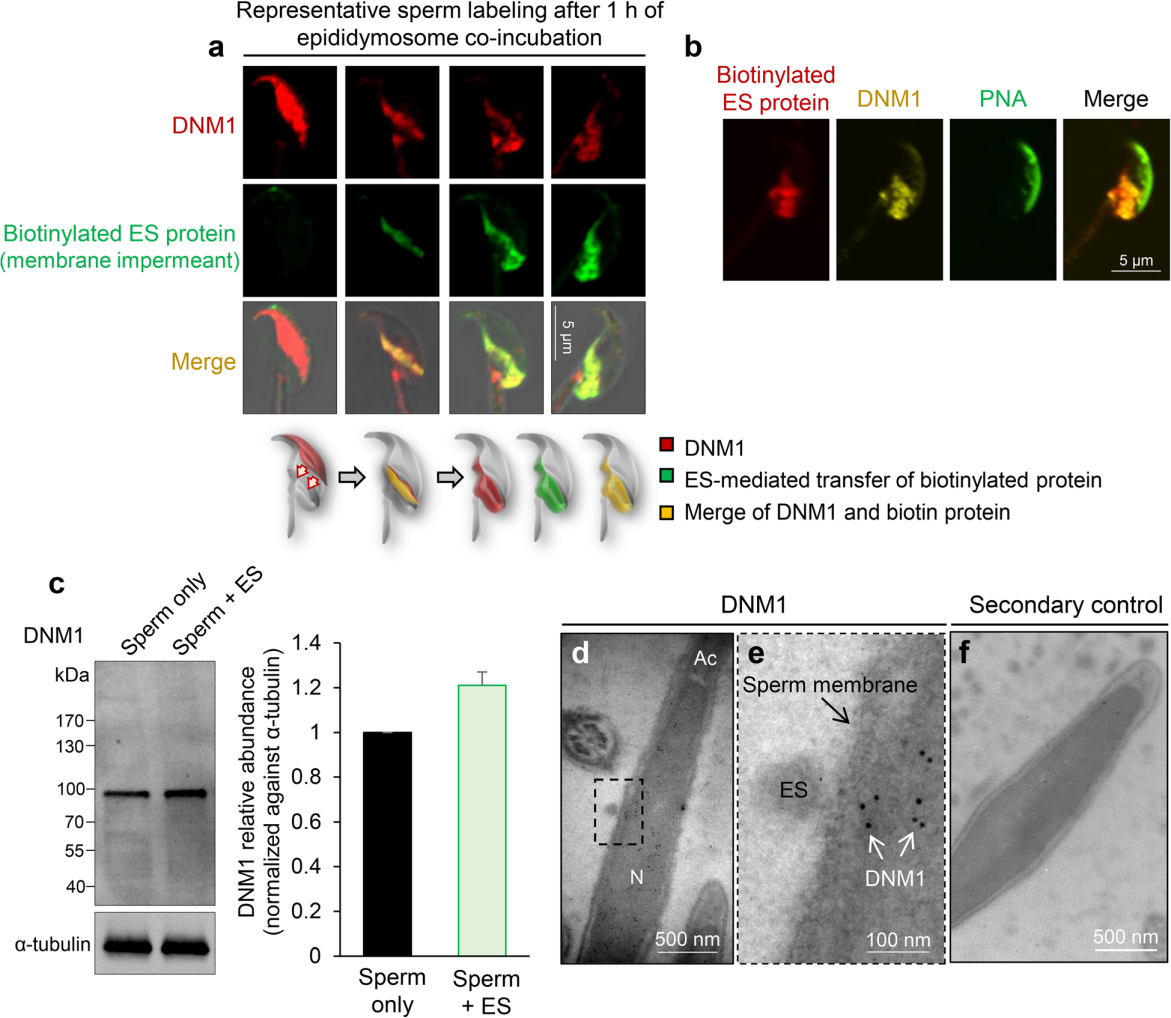
Analysis of the involvement of DNM1 in epididymosome-sperm interaction. **a** Spermatozoa were incubated with biotin (membrane impermeant) labeled epididymosomes for 1 h before being subjected to immunofluorescence detection of biotin (green) and DNM1 (red). Representative immunofluorescence images of the different sperm labeling patterns detected after this period of co-incubation, and a schematic model, are presented to illustrate an apparent relocation of endogenous sperm DNM1 to the post-acrosomal domain and an accompanying transfer of biotinylated epididymosome proteins to an equivalent region. **b** To preclude the possibility that these changes in the localization reflected an unmasking of an additional pool of DNM1 due to spontaneous loss of the acrosomal domain, triple immunofluorescence staining was applied to detect DNM1 (yellow), biotin (red), and the outer acrosomal membrane (PNA; green) in the same cells. **c** The relative abundance of DNM1 was quantified by immunoblotting of sperm homogenates in naïve cells (Sperm only) as well as those exposed to co-culture with epididymosomes (Sperm + ES). Band intensity was normalized relative to that of α-tubulin, with the sperm only control nominally set to a value of 1 (*n* = 3). Individual data points for each replicate are provided in Additional file 7: Raw data. **d**–**f** Immunoelectron TEM was utilized to localize DNM1 in spermatozoa within the lumen of caput epididymal tissue. **d** A representative image is shown, with **e** the inset focusing on a site in which epididymosome-sperm docking was apparent (i.e., boxed region in panel **d**). Such interactions were predominantly found in association with the membrane overlying the post-acrosomal sheath/posterior region of the caput sperm head and invariably, gold labeling depicting the localization of endogenous sperm DNM1 was detected in the vicinity of the epididymosome docking sites (white arrows). **f** The specificity of gold labeling was confirmed by the inclusion of secondary antibody only controls, which consistently failed to label spermatozoa or epididymosomes. N, nucleus; Ac, acrosomal domain; ES, epididymosome

*DNM1*: the DNM1 protein was exclusively localized to the peri-acrosomal domain of naïve caput spermatozoa as well as those sampled immediately after the introduction of epididymosomes (Fig. 6a). Unexpectedly, however, DNM1 was found to have undergone an apparent relocalization; initially to the SAR and thereafter to the post-acrosomal domain of sperm sampled at more advanced stages of co-incubation. Notably, the number of sperm experiencing these changes in DNM1 localization mirrored those that had incorporated biotinylated epididymosome proteins (data not shown), prompting us to investigate the co-localization of DNM1 and biotinylated proteins during representative stages of epididymosome protein transfer. With the exception of those cells in which DNM1 was restricted to the anterior (peri-acrosomal) domain of the sperm head (and consequently displayed minimal biotin labeling), this strategy revealed strong overlapping distribution of DNM1 and biotinylated epididymosome proteins (Fig. 6a). Illustrative of this, after 1 h of co-incubation with epididymosomes, ~ 3% and ~ 40% of spermatozoa displayed co-localization of DNM1 and biotin labeling within either the sub-acrosomal ring or the post-acrosomal domain of the sperm head, respectively. To discount the possibility that DNM1 under-representation in the peri-acrosomal domain was caused by the cells experiencing a premature or spontaneous loss of their acrosomal contents, triple immunofluorescence staining was applied to detect DNM1, biotin-labeled epididymosome proteins, and peanut agglutinin (PNA), a recognized marker of the outer acrosomal membrane (Fig. 6b). This analysis confirmed the co-localization of DNM1 and biotin-labeled protein in the post-acrosomal domain while PNA was clearly retained in the acrosomal domain, proving these cells possess an intact acrosome. As an additional control, we also investigated the relative levels of endogenous DNM1 versus those present in the cell after co-incubation with epididymosomes. Densitometric analysis on the resultant immunoblots revealed no significant difference in DNM1 levels in either cell population (Fig. 6c). Thus, despite the presence of DNM1 in mouse epididymosomes (Additional file 2: Figure S2), the modest levels these vesicles contain are unlikely to account for the altered profile of DNM1 labeling in sperm post-epididymosome incubation.

Having documented an apparent relocation of DNM1 to coincide with the site of epididymosome adhesion in the post-acrosomal domain, we next sought to strengthen the physiological relevance of this observation through the application of transmission immunoelectron microscopy to track DNM1 localization during epididymosome-sperm interactions in situ. As shown in Fig. 6, we consistently observed epididymosome docking to the post-acrosomal domain of the caput sperm head. Such events were commonly accompanied by immunogold labeling of endogenous sperm DNM1 within the vicinity of the membrane docking site. To preclude the possibility of non-specific labeling, sections were incubated with secondary antibody only revealing no appreciable staining of the spermatozoa (Fig. 6f).

**Fig. 7:**
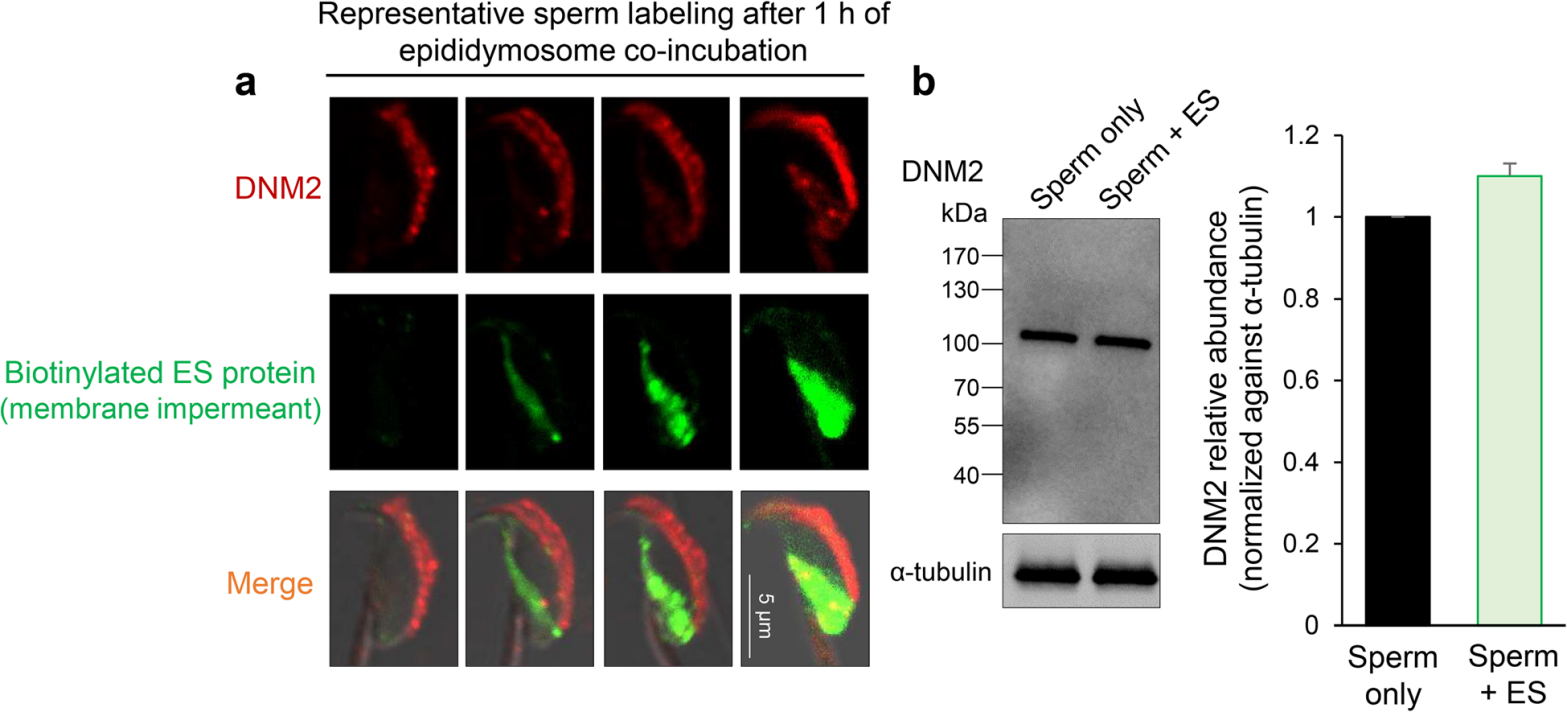
Analysis of the involvement of DNM2 in epididymosome-sperm interaction. **a** Spermatozoa were incubated with biotin (membrane impermeant)-labeled epididymosomes for 1 h before being subjected to immunofluorescence detection of biotin (green) and DNM2 (red). Representative immunofluorescence images of the different sperm labeling patterns detected after this period of co-incubation are provided to illustrate the labeling of DNM2 in the acrosomal domain and minimal co-localization with transferred biotinylated proteins. Indeed, only relatively weak DNM2 labeling was detected in the post-acrosomal domain of those cells that incorporated abundant biotinylated proteins. **b** The relative abundance of DNM2 was quantified by immunoblotting of sperm homogenates in naïve cells (Sperm only) as well as those exposed to co-culture with epididymosomes (Sperm + ES). For the purpose of comparing the relative abundance of DNM2, band intensity was normalized relative to that of α-tubulin, with sperm only control nominally set to a value of 1 (*n* = 3). Individual data points for each replicate are provided in Additional file 7: Raw data. These experiments were replicated three different times with each sample representing pooled material obtained from at least three mice

*DNM2* is similar to DNM1; endogenous DNM2 was also readily detected in the peri-acrosomal domain of caput spermatozoa (Fig. 7a). However, the DNM2 isoform did not appear to undergo any pronounced change in location upon co-incubation with epididymosomes (Fig. 7a). In this context, only weak DNM2 labeling was observed in the post-acrosomal domain coinciding with those cells in which a substantial amount of biotinylated protein transfer was detected; raising the prospect that this additional pool of DNM2 may have been transferred to the cells as part of the epididymosome cargo. However, despite the detection of DNM2 within caput epididymosomes (Additional file 2: Figure S2), densitometric analysis revealed only a modest, non-significant, increase in the abundance of DNM2 before and after epididymosome co-incubation (Fig. 7b).

### DNM1 relocation is linked to lipid raft association

**Fig. 8:**
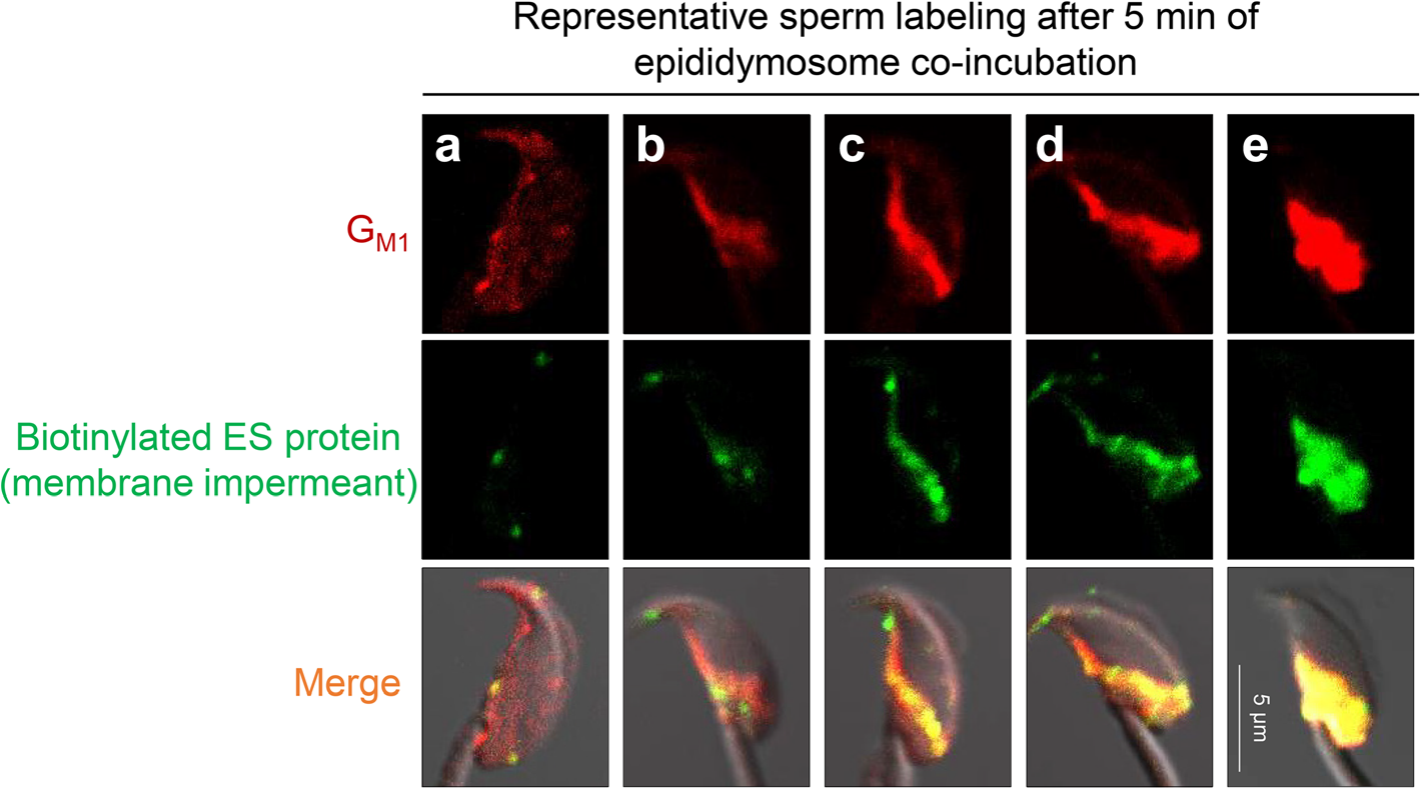
Lipid raft microdomains facilitate epididymosome-sperm interaction**.** Caput spermatozoa were co-cultured with biotinylated (membrane impermeant) epididymosomes for 5 min before being subjected to dual labeling for G_M1_ (lipid raft marker) and biotin. **a**–**e** This strategy confirmed that spermatozoa harbored the anticipated spatial profiles of transferred biotinylated protein (green) and revealed strong overlapping co-localization of G_M1_ (red) in these domains. Representative immunofluorescence images are presented to illustrate co-localization of G_M1_ and biotinylated protein in the SAR and post-acrosomal domain of the sperm head. These experiments were replicated three times with each sample representing pooled material (i.e., spermatozoa and epididymosomes) obtained from at least three mice

**Fig. 9:**
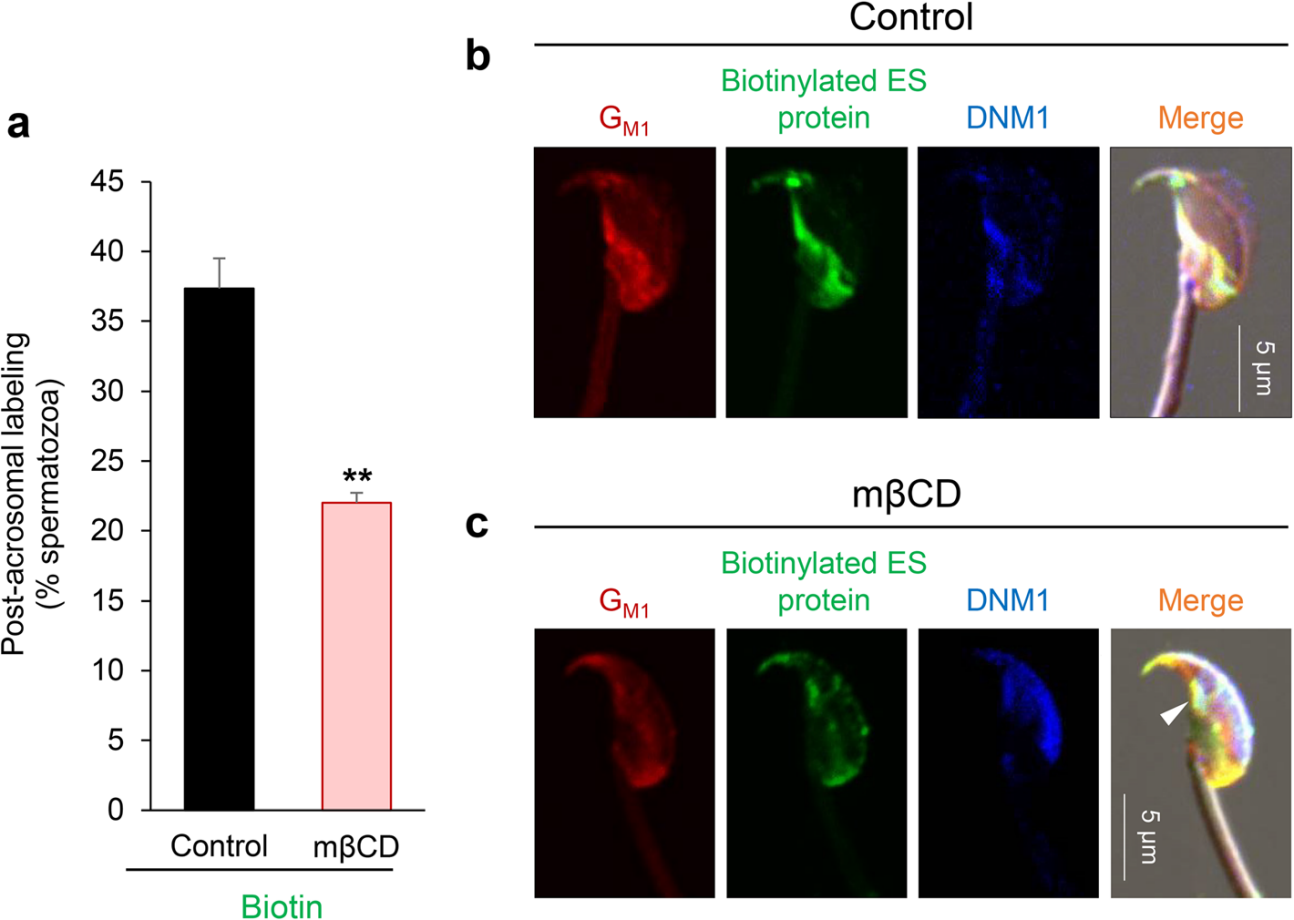
Disruption of sperm lipid rafts compromises the efficacy of DNM1 translocation and epididymosome-sperm interaction. To examine the role of lipid rafts in mediation of epididymosome-sperm interactions, cells were pre-treated with mβCD to sequester membrane cholesterol and disrupt lipid raft integrity. Thereafter, spermatozoa were incubated with biotinylated (membrane impermeant) epididymosomes for 1 h. Spermatozoa were then fixed and subjected to immunofluorescence detection. **a** A significant reduction in the number of cells with post-acrosomal biotin labeling was observed in spermatozoa pre-treated with mβCD vs those of untreated controls. Post-acrosomal labeling was assessed in a minimum of 100 cells per treatment group, with these experiments being replicated three times. Each replicate comprised pooled material from at least three mice. The results are presented as the mean ± S.E.M. **P < 0.01 compared to control. Individual data points for each replicate are provided in Additional file 7: Raw data. **b**, **c** Representative immunofluorescence images of triple stained caput spermatozoa: G_M1_ (red; lipid rafts), biotin (green), and DNM1 (blue). Compared to untreated control (**b**), mβCD treatment (**c**) elicited a loss of raft integrity with G_M1_ being heterogeneously dispersed throughout the sperm head. In these cells, DNM1 was mainly retained in the acrosomal domain, but did display a tendency to co-localize with G_M1_ and biotinylated proteins (white arrowhead)

To explore the mechanism(s) involved in the relocation of DNM1 to a position compatible with regulation of epididymosome interaction, we elected to focus on lipid rafts, specialized membrane subdomains that have been implicated in the compartmentalization of proteins required for epididymosome docking to the sperm surface [24, 26]. Strengthening the rationale for this approach, DNM contains a pleckstrin homology domain, which is commonly involved in the recruitment of proteins to specific membrane domains [27]. For these studies, we first characterized the localization of the abundant sperm lipid raft marker, G_M1_ ganglioside (via staining with Alexa Fluor 594 conjugated cholera toxin B subunit) [28], in naïve populations of caput spermatozoa as well as those exposed to in vitro epididymosome co-culture. This strategy revealed that lipid rafts were initially distributed throughout the head of caput spermatozoa, but that the pattern of G_M1_ localization was quite variable (Additional file 3: Figure S3). Notably, the subset of sperm presenting with diffuse labeling of G_M1_ throughout the whole head were generally refractory to the incorporation of biotinylated protein from epididymosomes (Fig. 8a). By contrast, we did document alternative G_M1_ labeling patterns, which were reminiscent of those observed for transferred epididymosomes proteins (Fig. 8b–e). Accordingly, strong overlapping co-localization of G_M1_ and biotinylated epididymosome proteins were detected in the SAR (Fig. 8c) extending distally into the post-acrosomal domain (Fig. 8e). Importantly, triple labeling of spermatozoa with G_M1_, streptavidin conjugated to Alexa Fluor 488, and anti-DNM1 antibodies also confirmed strong co-localization of their respective targets (Fig. 9b). Based on these data, we infer that the relocation of endogenous sperm DNM1 may be reliant on lipid raft association.

**Fig. 10:**
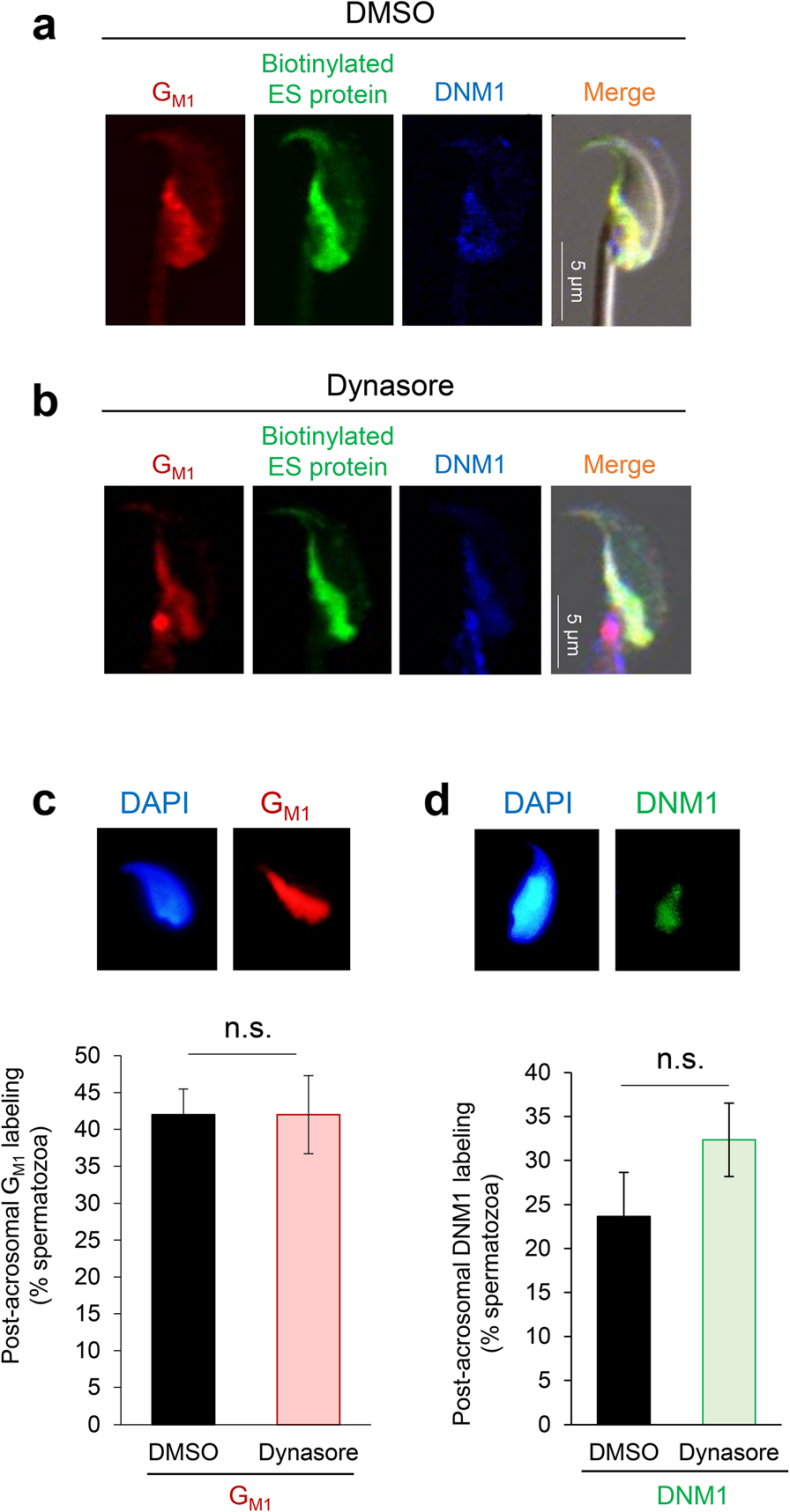
Pharmacological inhibition of dynamin does not compromise lipid raft or DNM1 translocation. To assess the impact of dynamin inhibition on lipid raft and DNM1 localization, caput spermatozoa were pre-treated with either the dynamin inhibitor, Dynasore, or the DMSO vehicle control. Thereafter, spermatozoa were incubated with biotinylated (membrane impermeant) epididymosomes for 1 h. Spermatozoa were then fixed and subjected to immunofluorescence detection. **a**, **b** Representative immunofluorescence images of triple stained caput spermatozoa are presented from DMSO and Dynasore-treated groups: G_M1_ (red; lipid rafts), biotin (green), and DNM1 (blue). However, due to the difficulty of counting the triple-stained cells, a subset of spermatozoa from each of the DMSO and Dynasore treatment groups were individually labeled for either **c** G_M1_ (red; lipid rafts) or **d** DNM1 prior to assessment of post-acrosome labeling. A minimum of 100 cells were assessed per treatment group, with these experiments being replicated three times. Each replicate comprised pooled material from at least three mice. The results are presented as the mean ± S.E.M. n.s. not significant compared to DMSO control. Individual data points for each replicate are provided in Additional file 7: Raw data

To test this hypothesis, spermatozoa were treated with methyl-β-cyclodextrin (mβCD) to sequester cholesterol and disrupt raft integrity [29] prior to their incubation with biotin-labeled epididymosomes. Triple immunofluorescence staining was then applied to detect DNM1, biotin-labeled protein, and G_M1_ distribution. In the control group, we routinely observed ~ 35% of the spermatozoa with post-acrosomal labeling for biotin (Fig. 9a), and each of these cells also displayed strong co-localization with DNM1 and G_M1_ in the same domain (Fig. 9b). By contrast, mβCD treatment disrupted lipid raft distribution, effectively preventing the accumulation of G_M1_ within the post-acrosomal domain (Fig. 9c). In parallel, we also documented a marked reduction in DNM1 relocalization, with the protein instead being retained predominantly within the anterior peri-acrosomal domain of mβCD-treated spermatozoa (Fig. 9c), suggesting that lipid raft integrity is important for DNM1 relocation. Such changes manifest in a significant (*P* < 0.01) reduction in the efficacy of biotinylated epididymosome protein transfer to the post-acrosomal domain compared to the untreated control group (Fig. 9a). Instead, biotinylated epididymosome proteins were distributed diffusely throughout the head of the mβCD-treated spermatozoa, effectively mirroring the localization of G_M1_ and thus adding further circumstantial evidence that lipid rafts do indeed facilitate epididymosome-sperm interactions. Unlike the dysregulation of lipid raft and DNM1 dynamics induced by mβCD, pre-treatment of caput spermatozoa with the dynamin inhibitor, Dynasore, prior to co-incubation with epididymosomes (as reported in Fig. 5) had no overt effect on the redistribution of either G_M1_ (Fig. 10b, c) or DNM1 (Fig. 10b, d) to the post-acrosomal domain of spermatozoa. Importantly, the specificity of triple immunofluorescence staining was confirmed by separate dual staining of two targets, including all combinations for DNM1, biotin, and G_M1_ (data not shown).

## Discussion

The epididymis fulfills an essential role in promoting sperm maturation and their subsequent storage via the creation of a complex intraluminal milieu, a key component of which are epididymosomes [30]. These small extracellular vesicles have attracted considerable attention owing to their important role in the transfer of fertility modulating proteins and regulatory classes of RNA to maturing spermatozoa [8]. To date, however, little is known of the mechanistic basis by which epididymosomes deliver their cargo to the maturing sperm cell. In previous work, we have identified that the post-acrosomal domain of mouse spermatozoa represents the predominant site for initial epididymosome-sperm interaction [19]. Here, we have confirmed and extended these observations via the use of a combination of lipophilic fluorophores and biotinylation reagents (both membrane permeant and impermeant) to differentially label epididymosome cargo. This strategy has provided evidence that epididymosome-sperm interactions are likely resolved into two sequential phases. Thus, a rapid vesicular docking, which is primarily restricted to the SAR/post-acrosomal domain of the sperm head, is followed by a transient vesicular fusion. The latter presumably facilitates cargo delivery prior to its bidirectional dispersal into the anterior region of the sperm head and mid-piece of the flagellum. Moreover, our data demonstrate that endogenous DNM1 is relocated to the post-acrosomal domain, through association with lipid rafts, to facilitate the transient fusion of epididymosome-sperm membranes.

Spermatozoa possess a unique membrane architecture, with the head of these cells being broadly divided into apical membrane, and post-acrosomal membrane, domains. Delimiting these two domains are topographical features known as the equatorial sub-segment (EqSS) and the SAR [31, 32]. Both of these structures have been implicated as specialized diffusion barriers, which limit lateral mixing of membrane components and thus establish heterogeneous molecular compartments in the sperm head with discrete roles in the fertilization cascade [33, 34]. Indeed, it has been suggested that the dense cytoskeletal structure of the SAR restricts anterior movement of membrane lipids into the apical plasma membrane domain. Moreover, the EqSS, which is dynamically assembled during sperm descent through the caput epididymis (i.e., increases in prevalence from ~ 30% of testicular sperm to ~ 78% of caput epididymal spermatozoa) [35], serves as a putative organizing center responsible for the assembly of multimolecular complexes that contribute to fusion competence in this area of the plasma membrane [35]. While we remain uncertain as to why epididymosomes may preferentially interact with the SAR, the imposition of EqSS and SAR may account for the subsequent segregation of epididymosome membrane proteins (i.e., those labeled with membrane impermeant biotin) into the anterior post-acrosomal domain; a phenomenon recorded in our study that also bears striking resemblance to that of independent evidence in the bovine model [18]. The post-acrosomal sheath is formed during the latter stages of spermatogenesis [36] as the spermatid head undergoes elongation and flattening, with its components providing structural reinforcement to maintain the acrosomal and nuclear domains. Notably, this region has also gained interest owing to its importance in initiating oocyte activation during mammalian fertilization [37, 38]. Thus, it has been argued that proteins selectively residing in the post-acrosomal sheath [e.g., post-acrosomal sheath protein WW domain-binding protein (PAWP)] enter the oocyte during fertilization and thereafter mediate meiotic resumption and oocyte activation [38, 39]. Such evidence provides a clear imperative for further investigation of the contribution of epididymosomes in establishing the proteomic specialization of both the plasma membrane overlying the post-acrosomal sheath as well as the cytoplasmic content of this domain.

Downstream of the rapid adhesion of epididymosomes to spermatozoa, which took place in as little as 1 min of co-culture, we recorded a more gradual, bidirectional transferal of biotinylated epididymosome proteins into the anterior region of the sperm head and the mid-piece of the flagellum. Notably, however, this phenomenon was restricted to the use of the membrane permeant biotin reagent, which would be expected to label both the epididymosome membrane and encapsulated cargo. This staining pattern was reminiscent of that achieved following co-incubation of mouse spermatozoa with epididymosomes preloaded with CFSE, an amine reactive dye that undergoes intracellular catalytic conversion into a highly fluorescent tracer with a propensity to form stable conjugates with proteins [7]. It is also more in keeping with independent reports that dye-labeled mouse epididymosomes can deliver the dye to the acrosomal domain of the head and mid-piece of the flagellum, albeit in caudal spermatozoa [24]. On the basis of these data, we infer that vesicular docking may precede internalization and redistribution of at least a portion of the epididymosome cargo, to their sites of action in the maturing sperm cell. While the precise details of how sperm achieve this internalization have yet to be completely resolved, evidence is mounting for a transient fusogenic mechanism between the respective epididymosome and sperm membranes. Indeed, there is general consensus that spermatozoa lack the machinery to participate in endocytosis, as is commonly witnessed in exosome-somatic cell interactions. Rather, high-resolution imaging techniques such as super-resolution structured illumination microscopy [40] and transmission immunoelectron microscopy have revealed compelling evidence for the formation of fusion stalk-like projections forming at sites of interaction between epididymosomes and spermatozoa. Moreover, proteomic analyses of epididymosomes, and spermatozoa themselves, have identified a myriad of complementary trafficking proteins [e.g., soluble *N*-ethylmaleimide-sensitive factor activating protein receptor (SNARE) proteins, Ras-like proteins and DNM] as might be expected in fusogenic competent vesicles/cells [16, 19, 22, 25].

One such family of proteins that we have investigated here is that of DNM, mechanoenzymes that have been implicated in a variety of vesicular trafficking pathways [25, 41]. While DNM has been best studied in the context of regulating clathrin-coated endocytosis [42], it has also been implicated in clathrin-independent pathways. These pathways include a “kiss and run” model that is compatible with the transient fusogenic mechanism proposed for epididymosome-sperm interaction. Within this model, DNM is held to polymerize into large oligomeric helices, which stabilize the formation of the vesicular fusion pores and thus regulate the release of their cargo [43]. Indeed, our recent ultrastructural data has revealed evidence for the formation of stalk-like projections at the site of epididymosome-sperm interaction [19], a classic template attracting DNM to polymerize into rings/helices [44]. As an important precedent for our own findings implicating DNM in epididymosome fusion, independent work has confirmed a role of DNM in regulating exosome interaction with recipient cells such as B lymphocytes [45]. In this context, pharmacological inhibition of DNM (i.e., Dynasore) led to an impressive ~ 88% reduction in exosome uptake [45]. Similarly, DNM has also been shown to exert influence over the exosome receptivity of hepatic stellate and placental trophoblast cells [46, 47], with its inhibition leading to a pronounced suppression of their downstream functionality. Also, compatible with our own data is the notion that DNM-mediated regulation of exosome interaction is intimately tied to lipid rafts and their associated proteins [45].

Indeed, one of the most intriguing findings of our study was the demonstration that DNM1 is repositioned to the post-acrosomal domain where epididymosomes interact with the spermatozoa during co-culture. Our collective evidence suggests that such relocation is mediated, at least in part, by DNM1 association with lipid rafts, with the depletion of membrane cholesterol causing a chain of lipid raft disruption, inhibition of DNM1 translocation, and reduction in the efficacy of epididymosome cargo incorporation into the sperm proteome. In a similar context, pharmacological inhibition of dynamin was also effective in reducing the number of recipient spermatozoa having epididymosome proteins distributed to the anterior region of the head and mid-piece of the tail. This treatment did not, however, influence the redistribution of either DNM1 or lipid rafts to the post-acrosomal domain, nor did it prevent the initial tethering of epididymosomes to this domain. These data reinforce the notion that lipid rafts provide the driving force for promoting DNM1 relocation, as opposed to vice versa, and that the early stages of epididymosome docking occurs independent of dynamin activity. A particular curiosity of this response is the fact that the caput spermatozoa sourced for in vitro co-culture had likely already encountered epididymosomes in vivo. How these cells retain DNM1 in their peri-acrosomal domain after isolation, yet reposition the protein following exposure to an exogenous supply of epididymosomes, remains a perplexing question for which we can only speculate on the answer.

One possible explanation for these dichotomous results is that DNM1 translocation is a dynamic event, such that the removal of spermatozoa from the epididymal luminal environment in which they are normally extremely highly concentrated, leads to an attendant loss of the stimulus that drives DNM1 localization. In seeking to reconcile this model, our transmission immunoelectron microscopy data revealed that DNM1 is almost exclusively localized to the post-acrosomal domain of spermatozoa in situ. Additionally, elegant studies by Jones and colleagues have shown that mammalian spermatozoa exhibit a mechanosensitive response that serves to concentrate important molecules to appropriate sites on the sperm surface [48]. Specifically, porcine spermatozoa experienced a phenomenon referred to as ‘contact induced coalescence’, whereby physical contact such as that experienced during sperm agglutination, promoted a rapid repositioning of lipid rafts; away from the apical ridge overlying the acrosome and clustering at the sites of contact [48]. By analogy, it is tempting to speculate that an equivalent diffusion of rafts may have been triggered via adhesion of excess epididymosomes to the post-acrosomal domain of cultured spermatozoa, bringing with them essential fusion machinery such as DNM1. However, the validity of this model awaits further investigation, as does the finding that DNM2 fails to undergo a similar relocation, instead remaining within the peri-acrosomal domain of caput spermatozoa during epididymosome co-culture.

These findings contrast the overlapping localization and functions of DNM1 and DNM2 that have been reported in somatic cells [49]. Nevertheless, despite sharing 80% sequence identity, DNM1 and DNM2 have previously been implicated in discrete functional roles within the male reproductive system. Thus, selective ablation of DNM2 leads to an age-dependent loss of spermatogonia in the mouse testis [50]. Similarly, DNM2 is under-represented and linked a reduced ability to complete acrosomal exocytosis, in poor quality human spermatozoa [3]. In both scenarios, DNM1 expression is unchanged, yet fails to compensate for the loss of DNM2. Based on these data, we infer that DNM1 may be involved in the regulation of epididymal maturation (i.e., regulating epididymosome-sperm interactions), while DNM2 participates in early germ cell development and the downstream functional activation of the mature spermatozoon. It will be of interest to determine whether this specificity is mediated by unique protein-interaction networks.

## Conclusions

In summary, this study has provided mechanistic insights into epididymosome-sperm interactions, revealing both the spatial and temporal specificity of this process. Such specificity is mediated, at least in part, via the action of lipid rafts owing to their ability to concentrate important molecules to sites of epididymosome interaction. Moreover, we have identified a novel role for vesicle trafficking machinery such as the DNM1 mechanoenzyme, which is likely to support/stabilize the formation of transient fusion pores compatible with the delivery of epididymosome cargo. As an important caveat, however, our study is based on the application of an in vitro co-culture system and we therefore encourage caution in direct extrapolation of our model of epididymosome-sperm interactions to the equivalent events occurring in situ within the epididymal lumen. Further studies aimed at overcoming these limitations and resolving how the proteomic inventory that epididymosomes convey to spermatozoa are able to modulate their function are now warranted if we are to realize the diagnostic and therapeutic potential of these insights.

## Methods

### Antibodies and reagents

Unless otherwise stated, chemicals were purchased from Sigma-Aldrich (St. Louis, MO, USA) or Thermo Fisher Scientific (Waltham, MA, USA) and were of molecular biology or research grade. Full details of the primary and secondary antibodies used throughout this study are reported in Additional file 4: Table S1.

### Mouse epididymosome isolation and characterization

Highly enriched populations of mouse caput epididymosomes were isolated and validated as previously described [7, 51]. Briefly, adult male mice (8–12 weeks old) were euthanized and immediately perfused with pre-warmed phosphate-buffered saline (PBS) to minimize the possibility of blood contamination. Caput epididymides were then removed, separated from fat and connective tissue, and rinsed with modified Biggers, Whitten, and Whittingham media (BWW; pH 7.4, osmolarity 300 mOsm/kg) [52] before being pooled; the number of pooled epididymides was adjusted in accordance with the downstream application (please see details provided in relation to each protocol), but generally incorporated tissue from at least three mice. Following incisions with a razor blade, luminal contents were allowed to disperse into the BWW media and filtered through a 70-μm membrane. The resultant suspension was sequentially centrifuged with increasing velocity at 4 °C (500×*g*, 5 min; 2000×*g*, 5 min; 4000×*g*, 5 min; 8000×*g*, 5 min; 17,000×g, 20 min; and finally 17,000×g for an additional 10 min) to eliminate all cellular debris. The supernatant was layered onto a discontinuous OptiPrep gradient (40%, 20%, 10%, and 5%), diluted with a solution of 0.25 M sucrose, 10 mM Tris. Density gradients were ultracentrifuged (160,000×*g*, 18 h, 4 °C) after which 12 equivalent fractions were collected and diluted in PBS before being subjected to a final ultracentrifugation (100,000×*g*, 3 h, 4 °C). Epididymosomes were subsequently collected from fractions 9 and 10 and characterized in accordance with the minimal experimental requirements for the definition of extracellular vesicles [53], featuring analysis of their purity and overall homogeneity, as previously described (Additional file 5: Figure S4) [7]. After assessment, pooled preparations of epididymosomes were apportioned between the different experimental treatment groups as described below.

### Transfer of epididymosome protein cargo to mouse spermatozoa

Following isolation, caput epididymosomes were resuspended in PBS. Two different biotin reagents were then applied to label either the subset of membrane-accessible epididymosome proteins (i.e., membrane impermeant EZ-Link sulfo-NHS-LC-Biotin, Thermo Fisher Scientific) or those residing in both the membrane and encapsulated within the epididymosome (i.e., membrane permeant EZ-Link BMCC-Biotin, Thermo Fisher Scientific) with the use of both biotin reagents conforming to manufacturer’s recommendations. Biotinylation reactions were conducted for 30 min at room temperature followed immediately by overnight incubation at 4 °C. As a vehicle control for the use of membrane permeant BMCC-biotin, a population of epididymosomes were prepared with an equivalent volume of vehicle [Dimethyl sulfoxide (DMSO)]. Following incubation, epididymosome suspensions were diluted into 50 mM glycine/PBS to quench the biotinylation reaction and excess biotin was removed via ultracentrifugation (100,000×*g*, 18 h, 4 °C). The resultant biotinylated epididymosome pellets were suspended in modified BWW in preparation for co-incubation with caput epididymal spermatozoa; isolated as previously described [54]. Treatment groups included spermatozoa pre-incubated with either: (i) 0.5 mM mβCD to sequester membrane cholesterol and thereby disrupt lipid rafts) [29] for 1 h, (ii) 100 μM Dynasore (inhibits DNM1 and DNM2 with equivalent efficacy) for 30 min, or (iii) 100 μM Dyngo-Ɵ (an inactive analogue of Dynasore) for 30 min. Unless otherwise stated, biotinylated epididymosomes were added at a ratio of 1:1 (i.e., pooled epididymosomes isolated from three mice were subdivided into three equivalent fractions, and one of these fractions was incubated with spermatozoa isolated from a single mouse). Co-incubations were conducted in an atmosphere of 5% CO_2_ using the conditions specified in each figure legend. After incubation, cells were washed three times by gentle centrifugation (500×*g*, 3 min) in modified BWW to remove any unbound or loosely adherent epididymosome, before been subjected to 4% paraformaldehyde (PFA) fixation (for immunofluorescent staining) or protein extraction (for silver stain or immunoblotting as previously described) [41]. Controls for these experiments included spermatozoa directly labeled with both biotin reagents (i.e., in the absence of any epididymosomes) to discriminate the specificity of epididymosome-mediated protein delivery, as well as spermatozoa incubated with unlabeled epididymosomes in order to control for the possibility of endogenous biotin expression.

### Transfer of lipophilic dyes between epididymosomes and spermatozoa

A PKH26 Fluorescent Cell Linker Mini Kit (MINI26, Sigma-Aldrich) was used to label epididymosome membranes. For this purpose, caput epididymosomes (from three mice) were resuspended in 0.5 ml Diluent C and incubated with PKH26 (2 μl dye diluted into 0.5 ml Diluent C, then mixed 1:1 with epididymosomes suspension) for 2 min at room temperature with gentle agitation, thereby achieving irreversible labeling of the epididymosome lipid bilayer. After incubation, excess PKH26 dye was quenched by adding 1 ml of 1% bovine serum albumin (BSA)/PBS, and suspensions were ultracentrifuged (100,000×*g*, 3 h, 4 °C) to pellet PKH26-labeled epididymosomes. Isolated caput epididymal spermatozoa were co-incubated with PKH26-labeled epididymosomes under identical conditions to those described for biotin-labeled epididymosomes. The spermatozoa were then split into two equivalent samples, which were subjected to either confocal time-lapse imaging or 4% PFA fixation (for preservation and later-stage imaging). Additional controls were included in which spermatozoa were either directly labeled with PKH26 or incubated with unlabeled, Diluent C-treated epididymosomes (negative control).

### Affinity and immunofluorescent labeling of spermatozoa

Following incubation, spermatozoa were preloaded with Alexa Fluor 594 conjugated cholera toxin B subunit at 37 °C for 30 min to selectively label the abundant sperm lipid raft marker, G_M1_ ganglioside (if applicable). After that, cells were fixed in 4% PFA, washed in 50 mM glycine/PBS, and settled onto poly-l-lysine-treated coverslips at 4 °C overnight. They were then permeabilized with ice-cold methanol for 10 min and blocked with 3% BSA in PBS at 37 °C for 1 h. Coverslips were then incubated with primary antibodies at 4 °C overnight (for specific dilution rates of all antibodies see Additional file 4: Table S1). After three washes in PBS, coverslips were incubated with appropriate secondary antibodies or streptavidin conjugated to Alexa Fluor 488 at 37 °C for 1 h. Following additional washes in PBS, cells were counterstained with FITC-conjugated PNA (1 mg/ml) at 37 °C for 15 min (if applicable). Coverslips were then mounted in 10% Mowiol 4–88 (Merck Millipore, Darmstadt, Germany) with 30% glycerol in 0.2 M Tris (pH 8.5) and 2.5% 1, 4-diazabicyclo-(2.2.2)-octane. Confocal microscopy (Olympus IX81) was used for detection of fluorescent-labeling patterns with settings for excitation and emission filters being provided in Additional file 6: Table S2.

### Electron microscopy

Mouse caput epididymal tissue was fixed in 4% (*w*/*v*) PFA containing 0.5% (*v*/v) glutaraldehyde. The tissue was then processed via dehydration, infiltration, and embedding in LR White resin. Sections (100 nm) were cut with a diamond knife (Diatome Ltd., Bienne, Switzerland) on an Ultracut S microtome (Reichert-Jung, Leica; Solms, Germany) and placed on 150-mesh nickel grids. For DNM1 detection, sections were blocked in 3% (*w*/*v*) BSA in PBS (30 min at 37 °C). Subsequent washes were performed in PBS (pH 7.4) containing 1% BSA. Sections were sequentially incubated with anti-DNM1 antibodies (overnight at 4 °C), and an appropriate secondary antibody conjugated to 10-nm gold particles (2 h at 37 °C). Labeled sections were then counterstained in 1% (*w*/*v*) uranyl acetate. Micrographs were taken on a JEOL 1200 EX II transmission electron microscope (JEOL, Japan) at 80 kV.

### Statistical analyses

All experiments were replicated a minimum of three times, with pooled samples of spermatozoa and epididymosomes having been obtained from at least three male mice. For the purpose of assessing biotin labeling profiles, ≥ 100 spermatozoa were counted in each sample through blind assessment (with treatment conditions having been replaced with a random number) and the corresponding percentage of cells with post-acrosomal domain or whole head/mid-piece labeling was determined. Densitometric analyses of immuno/affinity blots were conducted using ImageJ software (version ImageJ2) [55]. Graphical data are presented as mean values ± SEM, which were calculated from the variance between samples. Statistical significance was determined by using one-way ANOVA with a significance threshold of *P* < 0.05.

## Additional files

Additional file 1: **Figure S1.** Time-lapse imaging of epididymosome-mediated PKH26 uptake into mouse spermatozoa. Caput epididymal spermatozoa were incubated with PKH26 labeled epididymosomes and immediately subjected to confocal imaging on a heated stage (37 °C). The real-time transfer of PKH26 from epididymosomes to spermatozoa was captured at 4–5-min intervals, illustrating that transfer was initiated within the SAR prior to extending distally into the post-acrosomal domain; a pattern of labeling that was consistent with the observed for biotinylated epididymosome cargo. (TIF 632 kb)

Additional file 2: **Figure S2.** Detection of DNM1 and DNM2 within caput epididymosomes. (a) Prior to detection of DNM1 and DNM2 proteins, aldehyde/sulphate latex beads were used to concentrate caput epididymosomes amenable with downstream fluorescence imaging applications. Staining was observed using fluorescence microscopy with the specificity of antibody labeling being confirmed through the inclusion of bead only controls (beads without attached epididymosomes) and secondary only control (no primary antibodies). (b, c) Caput epididymosome lysates (Caput ES) were resolved by SDS-PAGE and immunoblotted with either DNM1 or DNM2 antibodies. Mouse brain lysates were used as a positive control for DNM1 and DNM2 detection. (TIF 1173 kb)

Additional file 3: **Figure S3.** Detection of G_M1_ labeling of mouse caput epididymal spermatozoa. Immunofluorescence detection of G_M1_ gangliosides (an abundant lipid raft marker) was facilitated by labeling of caput epididymal spermatozoa with Alexa Fluor 594 conjugated cholera toxin B subunit. (a–e) A myriad of fluorescence staining patterns for G_M1_ were observed using confocal microscopy and the dominant profiles are depicted. (TIF 562 kb)

Additional file 4: **Table S1.** Details of antibodies used throughout this study (DOCX 23 kb)

Additional file 5: **Figure S4.** Assessment of epididymosome purity. A suite of assays were employed to assess the enrichment of epididymosomes, including (a) quantitative assessment of the protein content in the 12 equal fractions recovered after density ultracentrifugation; (b) immunoblotting to detect the distribution of the epididymosome marker FLOT1 within each of the 12 fractions; (c) detection of FLOT1 in epididymosomes concentrated via adhesion to aldehyde/sulphate beads; and (d) TEM assessment of the ultrastructure of the epididymosome population isolated from the pooling of fractions 9 and 10. Based on this analysis, epididymosomes partitioning into fractions 9 and 10 were pooled and used throughout the reported studies. (TIF 1607 kb)

Additional file 6: **Table S2.** Excitation and emission wavelengths used for detection of the different combinations of fluorophores in this study. (DOCX 13 kb)

Additional file 7: Raw data. This file contains raw data with individual data points or replicates for Figs. 6, 7, 9, and 10 (i.e., those experiments in which *n* < 6). (XLSX 11 kb)

## Abbreviations

BSA: Bovine serum albumin
BWW: Biggers, Whitten, and Whittingham media
CFSE: Carboxyfluorescein diacetate succinimidyl ester
DMSO: Dimethyl sulfoxide
DNM: Dynamin
DNM1: Dynamin 1
DNM2: Dynamin 2
EqS: Equatorial segment
EqSS: Equatorial sub-segment
MFGE8: Milk fat globule-EGF factor 8 protein
mβCD: Methyl-β-cyclodextrin
PAWP: Post-acrosomal sheath protein WW domain-binding protein
PBS: Phosphate-buffered saline
PFA: Paraformaldehyde
PNA: Peanut agglutinin
SAR: Sub-acrosomal ring
SNARE: Soluble *N*-ethylmaleimide-sensitive factor activating protein receptor
sncRNA: Small non-coding RNA
