# Supplementary figures and images for: Mechanisms of tethering and cargo transfer during epididymosome-sperm interactions
Source: BMC Biol. 2019 Apr 18;17:35. doi: 10.1186/s12915-019-0653-5 (PMC6474069; doi:10.1186/s12915-019-0653-5)

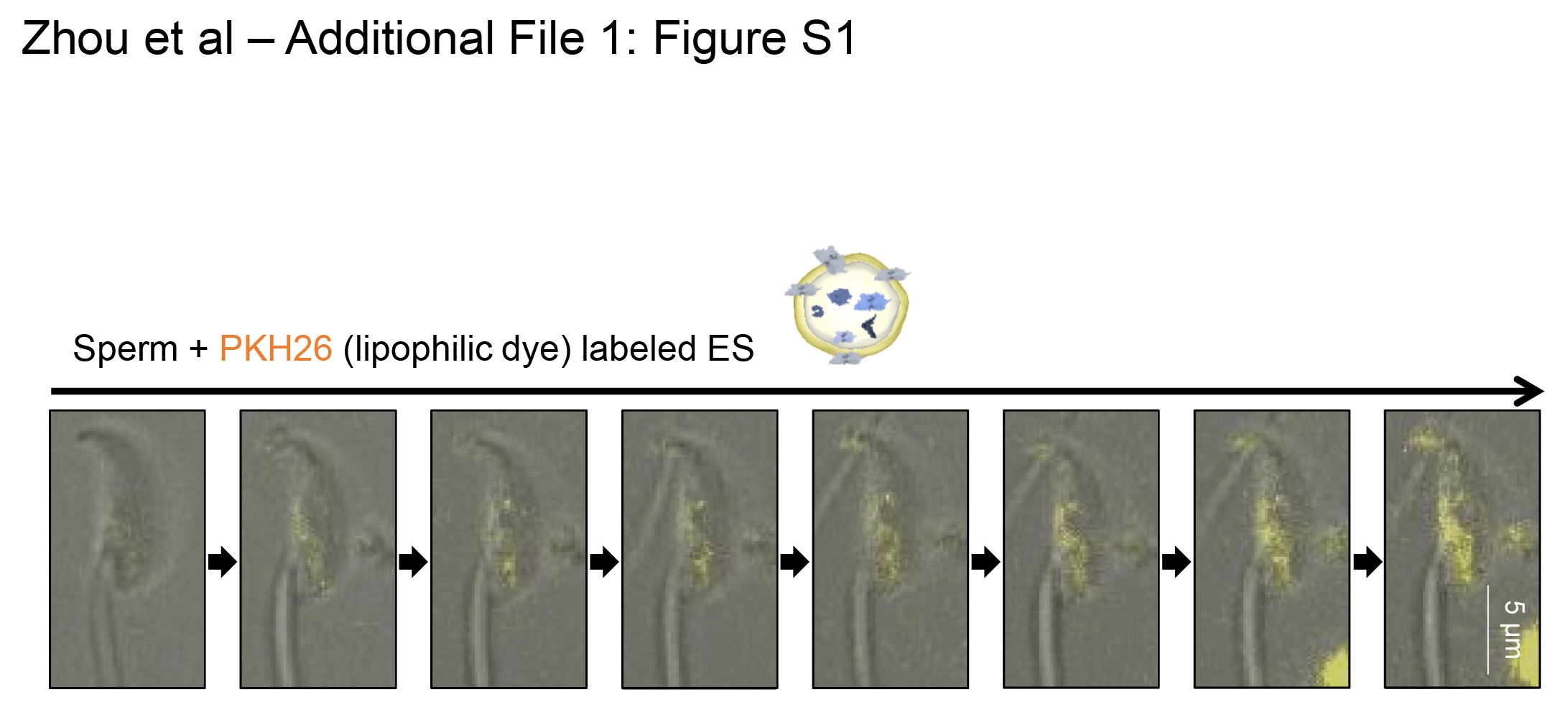

Supplement: Supplementary file 1 — Figure S1. Time-lapse imaging of epididymosome-mediated PKH26 uptake into mouse spermatozoa. Caput epididymal spermatozoa were incubated with PKH26 labeled epididymosomes and immediately subjected to confocal imaging on a heated stage (37 °C). The real-time transfer of PKH26 from epididymosomes to spermatozoa was captured at 4–5-min intervals, illustrating that transfer was initiated within the SAR prior to extending distally into the post-acrosomal domain; a pattern of labeling that was consistent with the observed for biotinylated epididymosome cargo. (TIF 632 kb) [file 12915_2019_653_MOESM1_ESM.tif]

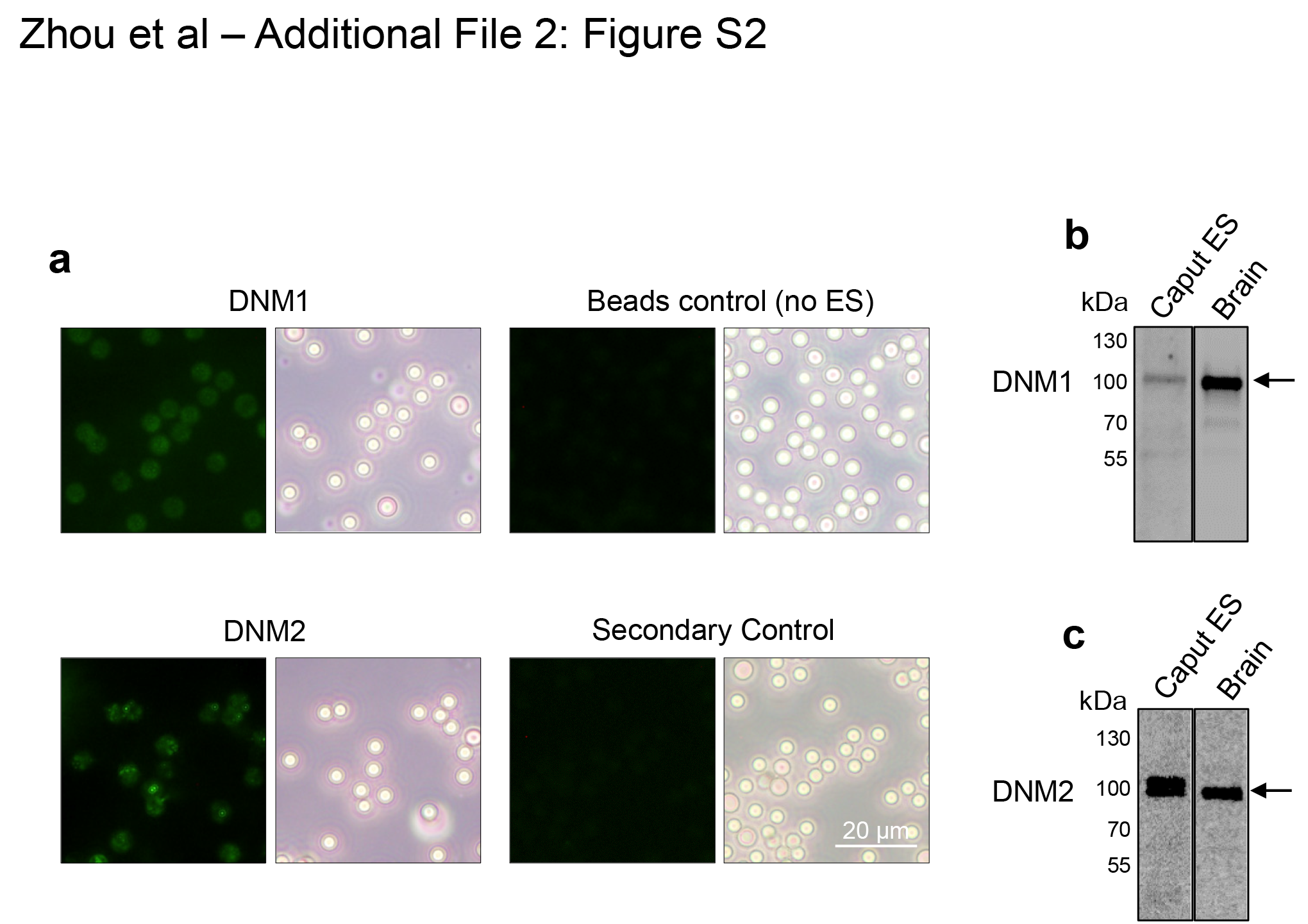

Supplement: Supplementary file 2 — Figure S2. Detection of DNM1 and DNM2 within caput epididymosomes. (a) Prior to detection of DNM1 and DNM2 proteins, aldehyde/sulphate latex beads were used to concentrate caput epididymosomes amenable with downstream fluorescence imaging applications. Staining was observed using fluorescence microscopy with the specificity of antibody labeling being confirmed through the inclusion of bead only controls (beads without attached epididymosomes) and secondary only control (no primary antibodies). (b, c) Caput epididymosome lysates (Caput ES) were resolved by SDS-PAGE and immunoblotted with either DNM1 or DNM2 antibodies. Mouse brain lysates were used as a positive control for DNM1 and DNM2 detection. (TIF 1173 kb) [file 12915_2019_653_MOESM2_ESM.tif]

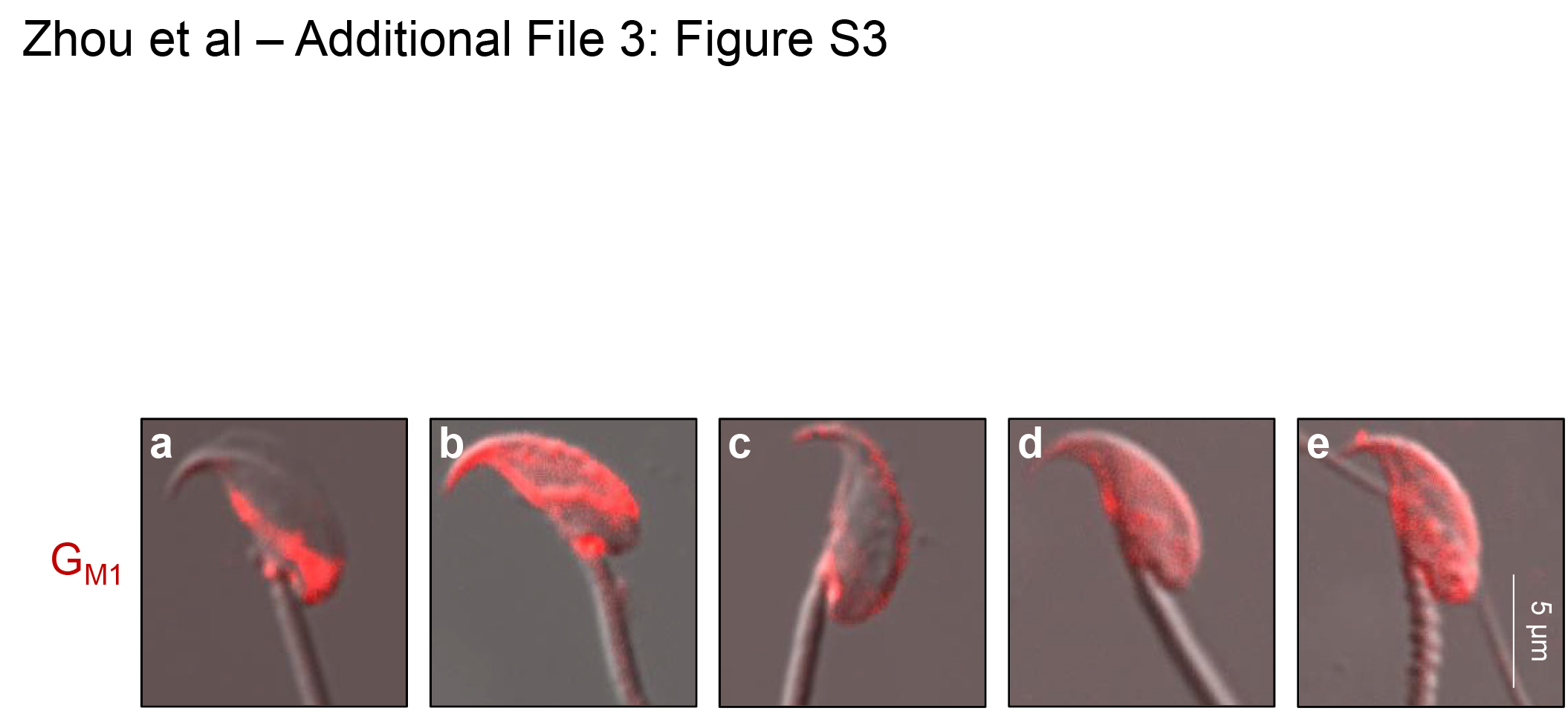

Supplement: Supplementary file 3 — Figure S3. Detection of GM1 labeling of mouse caput epididymal spermatozoa. Immunofluorescence detection of GM1 gangliosides (an abundant lipid raft marker) was facilitated by labeling of caput epididymal spermatozoa with Alexa Fluor 594 conjugated cholera toxin B subunit. (a–e) A myriad of fluorescence staining patterns for GM1 were observed using confocal microscopy and the dominant profiles are depicted. (TIF 562 kb) [file 12915_2019_653_MOESM3_ESM.tif]

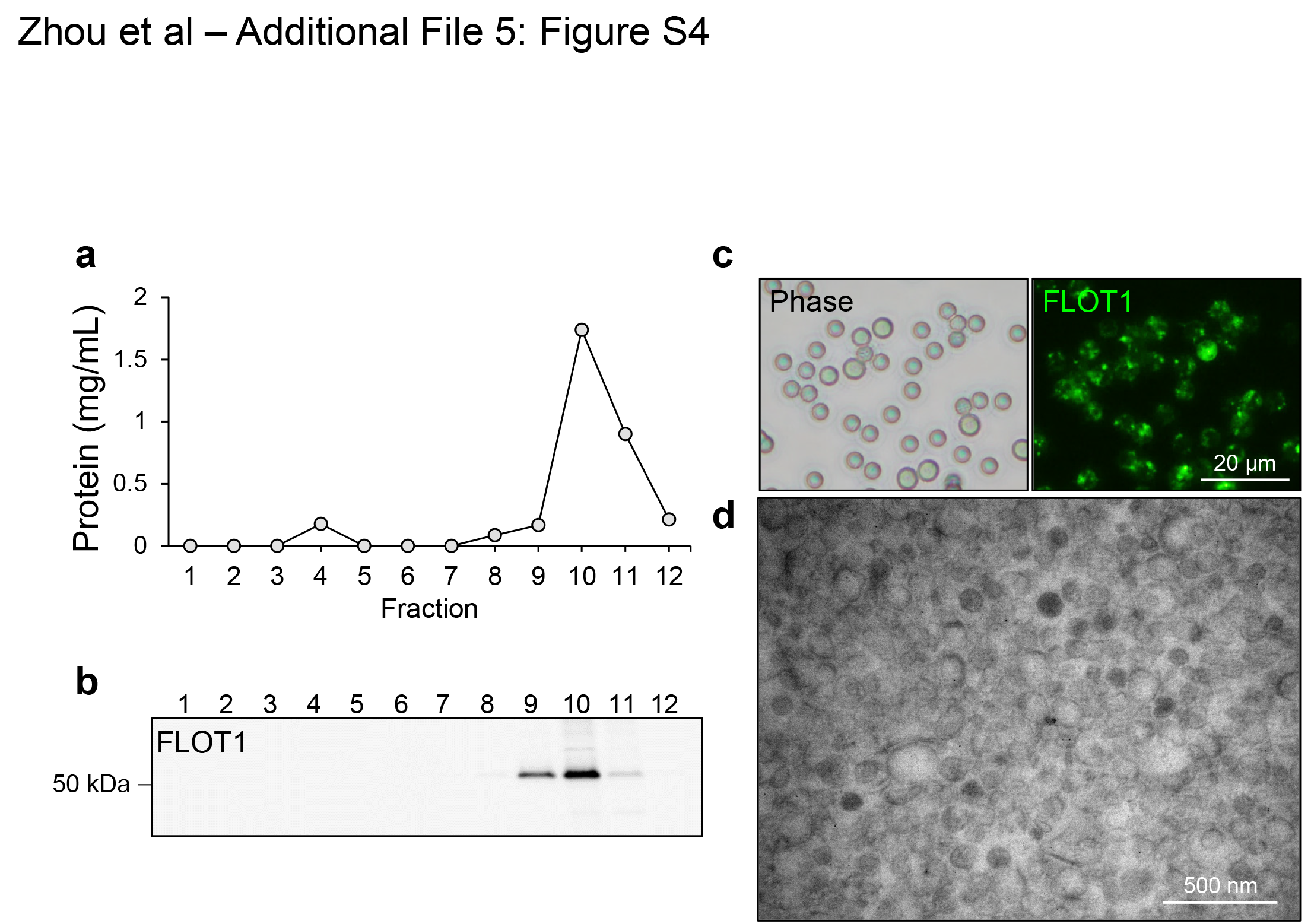

Supplement: Supplementary file 5 — Figure S4. Assessment of epididymosome purity. A suite of assays were employed to assess the enrichment of epididymosomes, including (a) quantitative assessment of the protein content in the 12 equal fractions recovered after density ultracentrifugation; (b) immunoblotting to detect the distribution of the epididymosome marker FLOT1 within each of the 12 fractions; (c) detection of FLOT1 in epididymosomes concentrated via adhesion to aldehyde/sulphate beads; and (d) TEM assessment of the ultrastructure of the epididymosome population isolated from the pooling of fractions 9 and 10. Based on this analysis, epididymosomes partitioning into fractions 9 and 10 were pooled and used throughout the reported studies. (TIF 1607 kb) [file 12915_2019_653_MOESM5_ESM.tif]
