# Supplementary material for: Mechanisms of tethering and cargo transfer during epididymosome-sperm interactions
Source: BMC Biol. 2019 Apr 18;17:35. doi: 10.1186/s12915-019-0653-5 (PMC6474069; doi:10.1186/s12915-019-0653-5)
Supplement: Supplementary file 4 — Table S1. Details of antibodies used throughout this study (DOCX 23 kb) [file 12915_2019_653_MOESM4_ESM.docx]

**Additional File 4: Table S1: Details of antibodies used throughout this study**

| **Antibody** | **Final concentration**  **(dilution of stock solution)^1^** | | | **Company** | **Catalogue N^o.^**  **(RRID)^2^** | **Batch N^o.^** | **Stock Concentration** |
| --- | --- | --- | --- | --- | --- | --- | --- |
| ***Primary antibodies*** | **IF** | **IB** | **EM** |  |  |  |  |
| **Dynamin 1** | 1 µg (1:50) | 1.5 µg (1:1000) | - | Abcam | ab108458 | GR107528-1 | 1 mg/mL |
| **Dynamin 1** | 1.25 µg (1:40) | - | 0.83 µg (1:20) | Abcam | ab13251  (AB_299794) | GR283893-2 | 1 mg/mL |
| **Dynamin 2** | 0.2 µg (1:50) | - | - | Santa Cruz Biotechnology | sc-6400  (AB_639943) | L0712 | 0.2 mg/mL |
| **Dynamin 2** | - | 0.4 µg (1:1000) | - | Thermo Fisher Scientific | PA5-19800  (AB_10983803) | QK2113152 | 0.27 mg/mL |
| **Flotillin 1** | 2 µg (1:100) | 1.5 µg (1:1000) | - | Sigma-Aldrich | F1180  (AB_1078893) | 124M4804V | 1 mg/mL |
| **α-Tubulin** | - | 2.85 µg (1:3000) | - | Sigma-Aldrich | T5168  (AB_477579) | 103M4773V | 5.7 mg/mL |
| **Cholera Toxin Subunit B 594 conjugate** | 0.125 µg (1:400) | - | - | Thermo Fisher Scientific | C34777 | - | 1 mg/mL |
| ***Secondary antibodies*** |  |  | |  |  |  |  |
| **Anti-rabbit Alexa Fluor 488** | 0.25 µg (1:400) | - | - | Thermo Fisher Scientific | A11008  (AB_143165) | 1678787 | 2 mg/mL |
| **Anti-goat Alexa Fluor 488** | 0.25 µg (1:400) | - | - | Thermo Fisher Scientific | A11055  (AB_142672) | 1369678 | 2 mg/mL |
| **Anti-goat Alexa Fluor 594** | 0.25 µg (1:400) | - | - | Thermo Fisher Scientific | A11058  (AB_142540) | 1180089 | 2 mg/mL |
| **Anti-mouse Alexa Fluor 594** | 0.25 µg (1:400) | - | - | Thermo Fisher Scientific | A11005  (AB_141372) | 1219862 | 2 mg/mL |
| **Anti-mouse Alexa Fluor 555** | 0.25 µg (1:400) | - | - | Thermo Fisher Scientific | A21422  (AB_141822) | - | 2 mg/mL |
| **Dylight-405 anti-mouse** | 0.375 µg (1:200) | - | - | Jackson ImmunoResearch | 715-475-150  (AB_2340839) | 130441 | 1.5 mg/mL |
| **Streptavidin, Alexa Fluor 633** | 0.25 µg (1:400) | - | - | Thermo Fisher Scientific | S21375  (AB_2313500) | - | 2 mg/mL |
| **Streptavidin, Alexa Fluor 488** | 0.25 µg (1:400) | - | - | Thermo Fisher Scientific | S11223  (AB_2336881) | 1733116 | 2 mg/mL |
| **HRP-Streptavidin** | - | N/A (1:1000) | - | Millipore | SA202 | ME9AN8751 | N/A |
| **Anti-rabbit HRP** | - | 1.3 µg (1:1000) | - | Millipore | DC03L  (AB_437852) | - | 0.13 mg/mL |
| **Anti-mouse HRP** | - | 0.15 µg (1:4000) | - | Santa Cruz Biotechnology | sc-2005  (AB_631736) | B1616 | 0.4 mg/mL |
| **Anti-mouse-Gold antibody** | - | - | N/A (1:10) | Sigma-Aldrich | G7652  (AB_259958) | 127k1520 | N/A |

^1^ IF, immunofluorescence; IB, immunoblot; EM, electron microscopy; -, not applicable; N/A, information not available from manufacturer

^2^ RRID: Research Resource Identifier
