# Supplementary material for: Mechanisms of tethering and cargo transfer during epididymosome-sperm interactions
Source: BMC Biol. 2019 Apr 18;17:35. doi: 10.1186/s12915-019-0653-5 (PMC6474069; doi:10.1186/s12915-019-0653-5)
Supplement: Supplementary file 6 — Table S2. Excitation and emission wavelengths used for detection of the different combinations of fluorophores in this study. (DOCX 13 kb) [file 12915_2019_653_MOESM6_ESM.docx]

| **Fluorescent dye combination** | **Excitation** | **Emission** |
| --- | --- | --- |
| Alexa Fluor 488 | 473 nm | 485-545 nm |
| Alexa Fluor 594 | 559 nm | 570-670 nm |
| Alexa Fluor 488 | 473 nm | 485-585 nm |
| Alexa Fluor 633 | 635 nm | 650-750 nm |
| FITC (PNA) | 473 nm | 485-545 nm |
| Alexa Fluor 555 | 559 nm | 570-625 nm |
| Alexa Fluor 633 | 635 nm | 655-755 nm |
| DyLight 405 | 405 nm | 425-460 nm |
| Alexa Fluor 488 | 473 nm | 485-545 nm |
| Alexa Fluor 594 | 559 nm | 575-675 nm |
| PKH26 | 559 nm | 570-670 nm |

**Additional File 6: Table S2: Excitation and emission wavelengths used for detection of the different combinations of fluorophores in this study.**
